# Mechanistic insights into the augmented effect of bone marrow mesenchymal stem cells and thiazolidinediones in streptozotocin-nicotinamide induced diabetic rats

**DOI:** 10.1038/s41598-018-28029-1

**Published:** 2018-06-29

**Authors:** Alaaeldin Ahmed Hamza, Ebtehal Mohammad Fikry, Wedad Abdallah, Amr Amin

**Affiliations:** 1 Hormone Evaluation Department, National Organization for Drug Control and Research (NODCAR), Giza, Egypt; 2 Department of Pharmacology, (NODCAR), Giza, Egypt; 3 Biology Department, College of Science, UAE University, Al-Ain, UAE; 4 Department of Zoology, Faculty of Science, Cairo University, Giza, Egypt

## Abstract

This study was designed to assess whether the protective effects of bone marrow-derived mesenchymal stem cells (MSCs) against diabetes could be enhanced by pioglitazone (PIO), a PPARγ agonist. Combined MSCs and PIO treatments markedly improved fasting blood glucose, body weight, lipid profile levels, insulin level, insulin resistance, β cell function. Those protective effects also attenuated both pancreatic lesions and fibrosis in diabetic rats and decreased the depletion of pancreatic mediators of glycemic and lipid metabolism including peroxisome proliferator-activated receptor alpha (PPARα), PGC-1α, GLP-1 and IRS-2. Cardiac biogenesis of diabetic groups was also improved with MSCs and/or PIO treatments as reflected by the enhanced up-regulation of the expressions of cardiac IRS1, Glucose transporter 4, PGC-1, PPARα and CPT-1 genes and the down-regulated expression of lipogenic gene SREBP. The combination of MSCs and PIO also potentiated the decrease of abnormal myocardial pathological lesions in diabetic rats. Similarly, the inhibitory effects of MSCs on diabetic cardiac fibrosis and on the up regulations of TGF-β, collagen I and III gene expressions were partial but additive when combined with PIO. Therefore, combined therapy with PIO and BMCs transplantation could further potentiate the protective benefit of MSCs against diabetes and cardiac damage compared to MSCs monotherapy.

## Introduction

Type 2 diabetes mellitus (T2DM) is the most common form of diabetes representing over 90% of all current diabetic cases. Diabetic cardiomyopathy is a major complication and the main cause of mortality among diabetic patients^1^. Patients with T2DM have a significantly higher risk of developing cardiovascular disease namely myocardial infarction, heart failure, and stroke^2^. The principle etiologies of T2DM incorporate insulin resistance in target tissues, relatively insufficient secretion of insulin, and subsequent decline of pancreatic β-cell function^3,4^. Multiple factors contribute to the development of cardiac dysfunction in diabetes including: alteration in lipid and glucose metabolism inside cardiomyocytes, induced oxidative stress, chronic inflammation, collagen deposition and apoptosis^5–7^. Insulin resistance in myocardium contributes to the adverse left ventricular remodeling and mitochondrial dysfunction leading to repression of insulin signaling pathways or glucose transporters (GLUTs; such as GLUT1 and GLUT4) in myocardium-mediated glucose transport^8^.

Metabolic dysregulation occurring in the heart of diabetic patients involves derangements in the activity of peroxisome proliferator-activated receptors (PPARs), PPAR gamma coactivator 1-alpha (PGC-1α), AMP-activated protein kinase (AMPK) and nuclear factor-kB (NF-κB). Consequently, oxidative stress, chronic inflammation, fibrosis, and cell death can be induced and eventually contribute to and exacerbate diabetic cardiomyopathy^4,6,9^. Heart under diabetes, and insulin resistance reduces glucose uptake and utilization and increases FA oxidation. This induced-modification of energy metabolism occurs via the reduced expression of GLUT4 and the dysregulation in the activity of both PPARs and PGC-1α signaling targets. Thus, targeting energy metabolism via improving glucose oxidation and/or fatty acid oxidation is a promising therapeutic strategy in the treatment of heart failure^9,10^.

PPARγ, the molecular target of the thiazolidinediones, is especially involved in the regulation of insulin sensitivity, inflammation, FA storage, and glucose metabolism. Thus, PPARγ represents an interesting pharmacological target which is able to simultaneously modulate several of the underlying pathologies of diabetes and metabolic syndromes^11^. Pioglitazone (PIO) is a thiazolidinedione drug and a PPAR γ agonist that acts as an insulin sensitizer, which has been used to treat T2DM for over a decade^7^ and has been shown to reduce cardiovascular events^12^. PIO has pleiotropic effects on insulin secretion, lipid and adipose tissue metabolism, body fat distribution and vascular endothelial function^6,7,12^. It was shown to effectively attenuate oxidative stress, inflammation and apoptosis^6,7^. Treatment of DM/obese rats with PIO increased the serum concentration of adiponectin and induced activation of AMPK in the left ventricle. Those effects were associated with attenuation of cardiac injury, fibrosis, and dysfunction as well as of cardiac oxidative stress and inflammation^13^.

Mesenchymal stem cells (MSCs) are one of the most important multipotent adult stem cells. Judged by their abilities to differentiate into any type of cells in damaged tissues, modulate their local environment, activate endogenous progenitor cells, and secrete various factors, MSCs seem to be a quite promising treatment of diabetes^14,15^. Recent studies have indicated that MSCs could potentially exert anti-diabetic effects, which resulted in the partial recovery of pancreatic islet, increased blood insulin secretion, and correction of hyperglycemia^16^. In animal models, MSCs were successfully used in STZ-induced diabetes both in rats and mice^16,17^. In diabetic NOD mice, the administration of a single dose of adult MSCs had neither curative effect on pancreatic regeneration^18^ nor on blood glucose improvement^19^. Another study found that MSC infusion restored the immune balance and increased the production of pancreatic islets from endogenous cells. Blood glucose levels fell in the MSC-treatment group, but did not reach normal levels^16^. In a previous study, the improvement in glucose control and the gain in body weight were not associated with an increase neither in leptin nor in insulin plasma levels^16^. Moreover, transplantation of one dose of MSCs only exhibited short-term effects and failed to restore normoglycemia in type two diabetic rat model induced by high-fat diet and STZ to rats^17^^,^^20^. They verified that multiple MSC infusions could reverse hyperglycemia and restore injured pancreatic islets. It has also been proposed that hyperglycemia in itself may be toxic and could prevent regeneration of endogenous pancreatic stem cells. Additionally, MSCs-therapy improved cardiac function in an experimental model of dilated cardiomyopathy through anti-apoptotic, anti-inflammatory mechanisms^21^. MSCs was also reported as a promising therapeutic strategy for acute myocardial infarction^22^. Moreover, the co- administration of PIO with MSCs showed significant superior improvement of heart function of rats with myocardial infraction^23^ and attenuated diabetic cardiomyopathy and mitochondrial dysfunction in type one diabetic rats^24^.

The aim of this study was to determine whether the protective effects of bone marrow-derived MSCs (BM-derived MSCs) in T2DM could be enhanced, and the cardiac complications could be attenuated, by combining MSCs treatment with the PIO in type 2 diabetic rats.

## Results

### Characterization of rat BM-MSC

**Figure 1:**
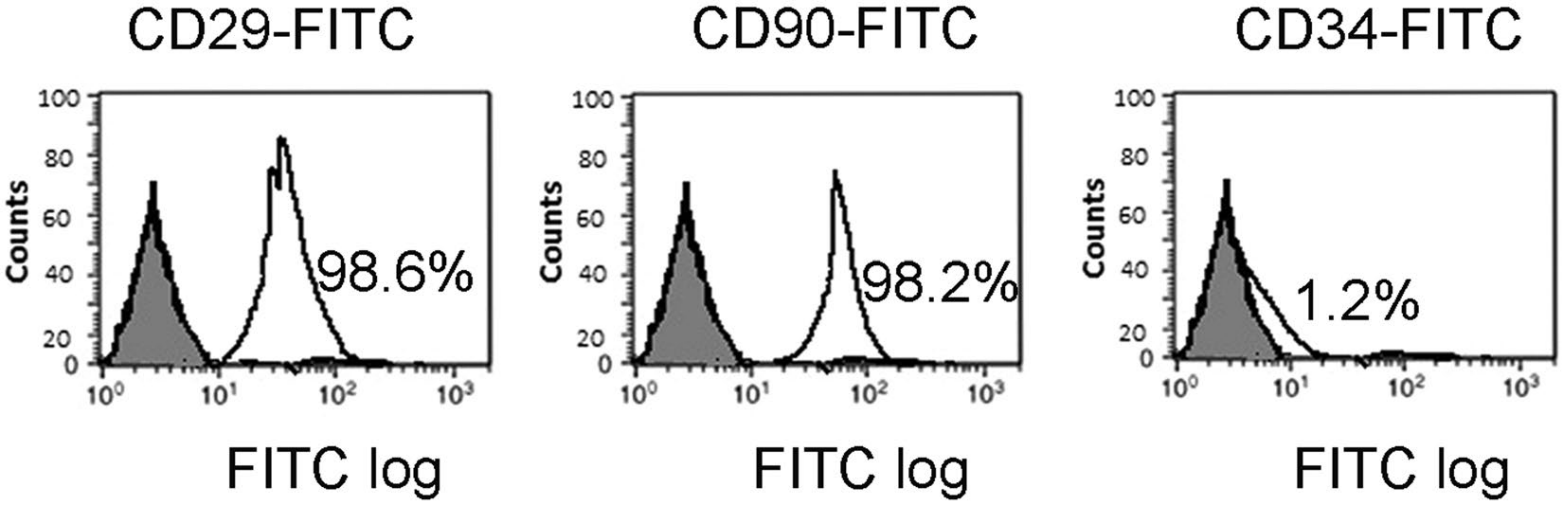
Characterization of BM-derived MSCs. Surface expression of CD29, CD90 and CD 34 of rat MSCs were monitored by FACS analysis. Percentage of cells expressing markers CD29, CD90 and CD34 were 98.6, 98.2, and 1.2%, respectively. FITC, fluorescein isothiocyanate.

BM-derived MSCs from Wistar rats were adherent cultured with a typical fibroblast-like morphology. FACS analysis showed that MSCs were strongly positive for CD29 (98.6%) and CD90 (98.2%) and negative for CD34 (1.2%) (Fig. 1).

### Effects of MSCs, PIO and their combination on physiological and biochemical parameters of STZ-NA- induced diabetic rats

**Figure 2:**
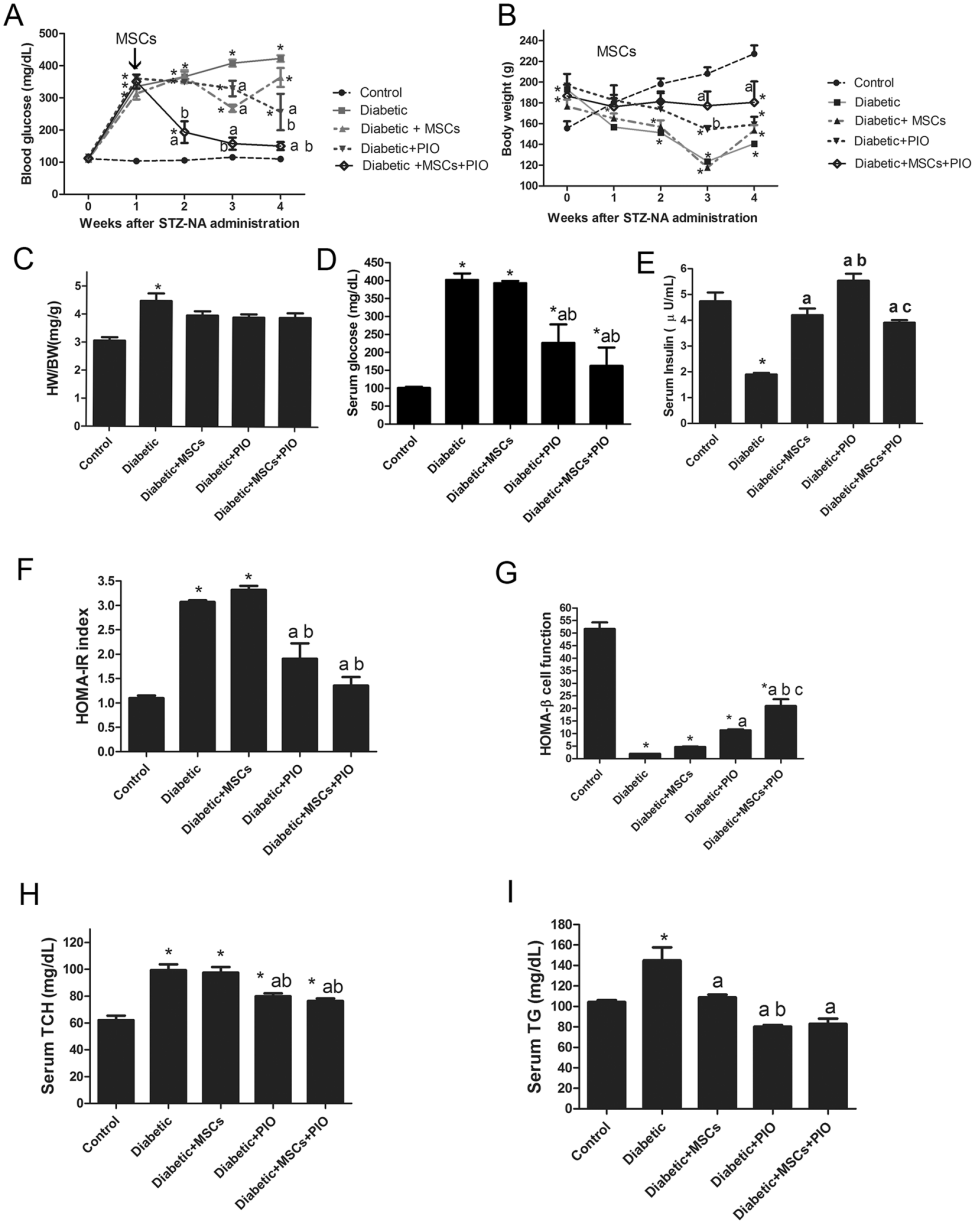
Effects of MSCs, PIO and their combination on physiological and biochemical parameters of STZ-NA-induced diabetic rats. Blood glucose (**A**), Body weight (**B**), HW/WT ratio (**C**), fasting serum glucose (**D**), fasting serum insulin (**E**), HOMA-IR index (**F**), HOMA-β index (**G**), TCH (**H**) and TG (**I**) in diabetic rats. All the experiments were repeated 2 times and all data are presented as Mean ± SEM (n = 6–8 animals per group). *P < 0.05 vs. control; a P < 0.05 vs. diabetic group; b P < 0.05 vs. Diabetic + MSCs group; c P < 0.05 vs. Diabetic + PIO group.

Diabetic rats exhibited increased fasting blood glucose levels (Fig. 2A), combined with gradual decrease in body weight (Fig. 2B) began after one week of diabetic induction. Moreover, most rats showed typical characteristic symptoms of diabetes including polyphagia, polydipsia and polyuria. Treatment with MSCs alone showed a transiently decrease of blood glucose level began after 2 week of diabetes induction and became significant at 3 week of diabetes induction and then began to increase again until week 4 without any improvement in body weight. PIO showed a transiently significant decrease of blood glucose level and body weight after 3 and 4 weeks of diabetes induction with transient improvement in body weight after 3 weeks of diabetes induction. After one week of diabetes, combination treatment with MSCs + PIO treatment showed significant decrease in fasting blood glucose levels with consistent increase in body weight comparing with Diabetic, Diabetic + MSCs, or Diabetic + PIO groups. These effects were sustained until 4 weeks. (Fig. 2A,B). Diabetic animals also had significantly increased in HW/BT ratio (Fig. 2C). Diabetic group also showed significantly decrease in serum insulin levels and HOMA- β cell function index (Figure E & G) with concomitant increase in serum glucose (D), HOMA- IR index (F), TCH and TG levels (Fig. 2D,F,H and I) compared with control group. Although MSCs treatment had no improvement in HOMA-IR index and HOMA-β cell function index or serum TCH level in diabetic rats after 4 weeks of diabetes compared to the control, serum glucose, TG and insulin levels or HW/BT ratio were significantly improved in Diabetic + MSCs group compared to the control rats. Both treatments with PIO alone or with MSCs and PIO improved serum TG, TCH levels, HOMA-IR index and HOMA-β cell function index comparing with diabetic and Diabetic + PIO groups. Interesting, HOMA-β cell function index in MSCs and PIO group was also significantly higher compared to Diabetic and Diabetic + MSCs groups.

### MSC, PIO and their combination reduced histopathological lesions of pancreas and in genes related to insulin sensitivity and islet function in diabetic rats

**Figure 3:**
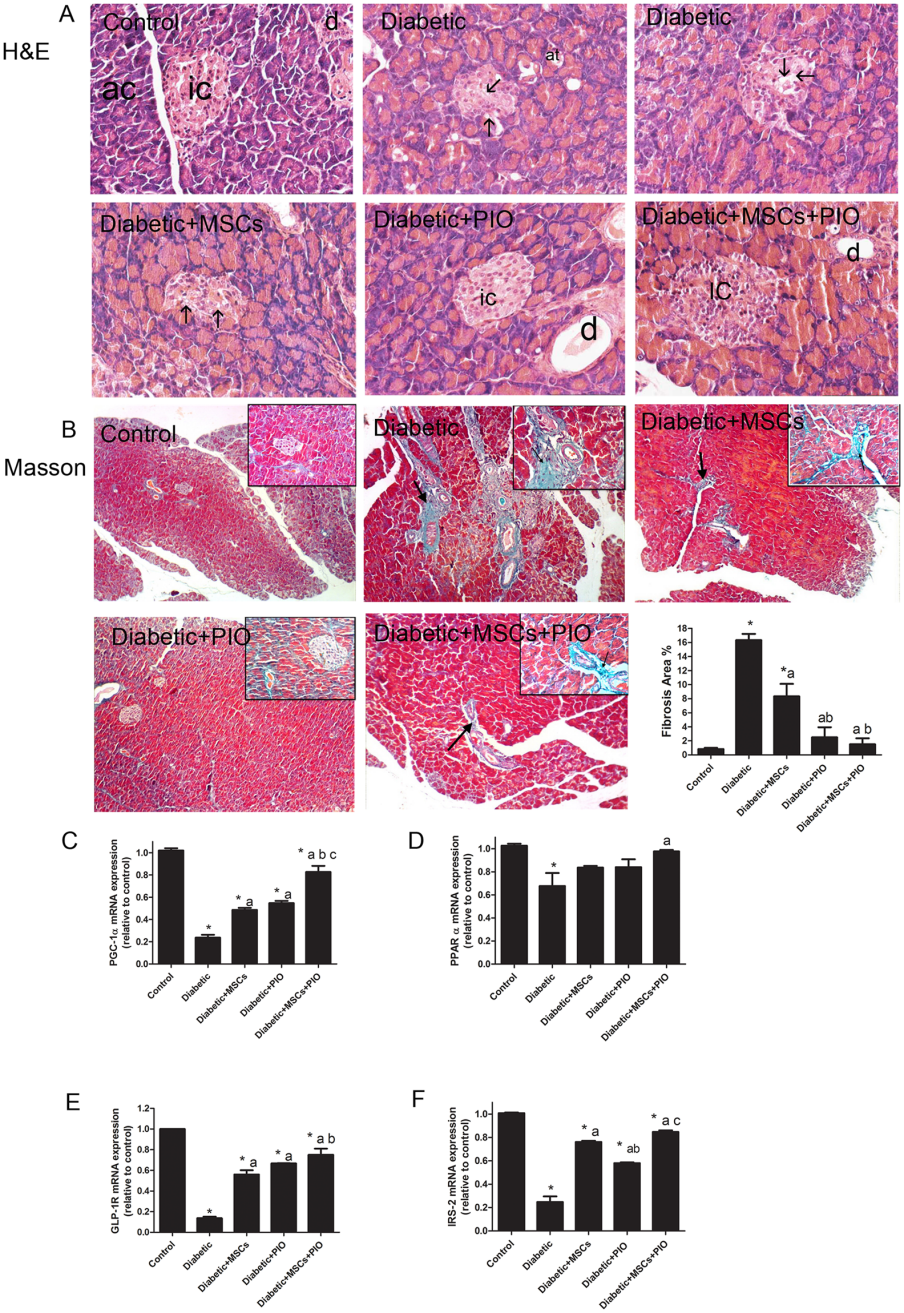
Effects of MSCs, PIO and their combination on pancreatic histopathological lesions and on genes related to insulin sensitivity and islet function in diabetic rats. (**A**) H&E stained sections of the different groups (400x). Control group showing islets of Langerhans (ic) formed of compact spherical masses of pale staining cells surrounded by pancreatic acini (ac) the cells are arranged in cords and separated by network of blood capillaries. Diabetic groups showed pancreatic lesion such as severe vacuolization of pancreatic islets (arrow, score 3), mild dilatation of pancreatic ducts (d, score 2). In MSCs and PIO treated diabetic rats the histological lesions were greatly reduced and were scored 1 for vacuolization of islets (arrow), and dilatation of pancreatic ducts. In diabetic groups treated with MSCs + PIO showed less pathological changes with score 0. (**B**) Masson staining (100x) showed increase in collagen fibers (arrow) around the acini and blood vessels and ducts, upper right inset depicts a higher magnification view (400x). In the diabetic group, collagen sheets were markedly thickened with more intense blue color after Masson staining around pancreatic blood vessels, ductules and acini. MSCs and PIO showed fewer collagen fibers around blood vessels, pancreatic ductules and acin as compared to diabetic group. Treatment with MSCs and PIO completely reduced collagen and showed normal distribution on collagen around blood vessels and acini. Pancreatic mRNA levels of PGC-1α (**C**), PPAR α (**D**), GLP-1R (**E**) and IRS-2 (**F**) were examined by RT-PCR analysis. All the experiments were repeated in 2 times and all data are presented as Mean ± s.e.m (n = 3 animals per group). *P < 0.05 vs. control; a P < 0.05 vs. diabetic group; b P < 0.05 vs. Diabetic + MSCs group; c P < 0.05 vs. Diabetic + PIO group; c P < 0.05 vs. Diabetic + PIO group.

To evaluate the effect of PIO and MSCs in morphology and function of pancreas in diabetic rats, we assessed the pancreatic pathologic changes by H&E and Masson staining and analyzed the pancreatic expressions of genes related to insulin sensitivity, islet regeneration and function such as PGC-1α, PPAR α, GLP-1R, and IRS-2 (Fig. 3).

Pancreas of the control rats showed normal lobular histological structure of pancreatic acini and round islet of Langerhans as shown in Fig. 3A. Pancreatic section from diabetic group displayed severe degeneration and vacuolation of pancreatic islets with dilatation of pancreatic duct. Pancreatic islets were largely reduced in size with a decrease in cellular density. Although the treatment of diabetic rats with either MSCs or PIO restored normal histology of pancreatic islets that were associated with less vacuolation, normal-sized islets were not restored. Interestingly, combination of MSCs and PIO treatment markedly reduced these lesions in pancreatic tissue of diabetic rats, and many islets showed an increase in cellular density with no vacoulation. Masson staining (Fig. 3B) showed increase in collagen fibers around the acini and blood vessels and ducts in diabetic pancreas. MSCs and PIO showed fewer collagen fibers around blood vessels and pancreatic acini and ducts as compared to diabetic group. Treatment with MSCs and PIO completely reduced collagen in pancreas and showed normal distribution on collagen around blood vessels and ducts (Fig. 3A,B). Furthermore, pancreatic mRNA levels of PGC-1α (Fig. 2C), PPAR α (Fig. 3D), GLP-1R (Fig. 3E) and IRS-2 (Fig. 3F) were all significantly decreased in diabetic rats. In contrast, pancreatic mRNA levels of PGC-1α, GLP-1R and IRS-2 were significant induced in MSCs + diabetic or PIO + diabetic groups as compared to diabetic group. The treatment with both MSCs + PIO further elevated pancreatic mRNA levels of those genes, compared to Diabetic + MSCs, or Diabetic + PIO groups.

### Effects of MSCs, PIO and their combination on genes related to glucose and lipid uptake and metabolism in hearts of diabetic

**Figure 4:**
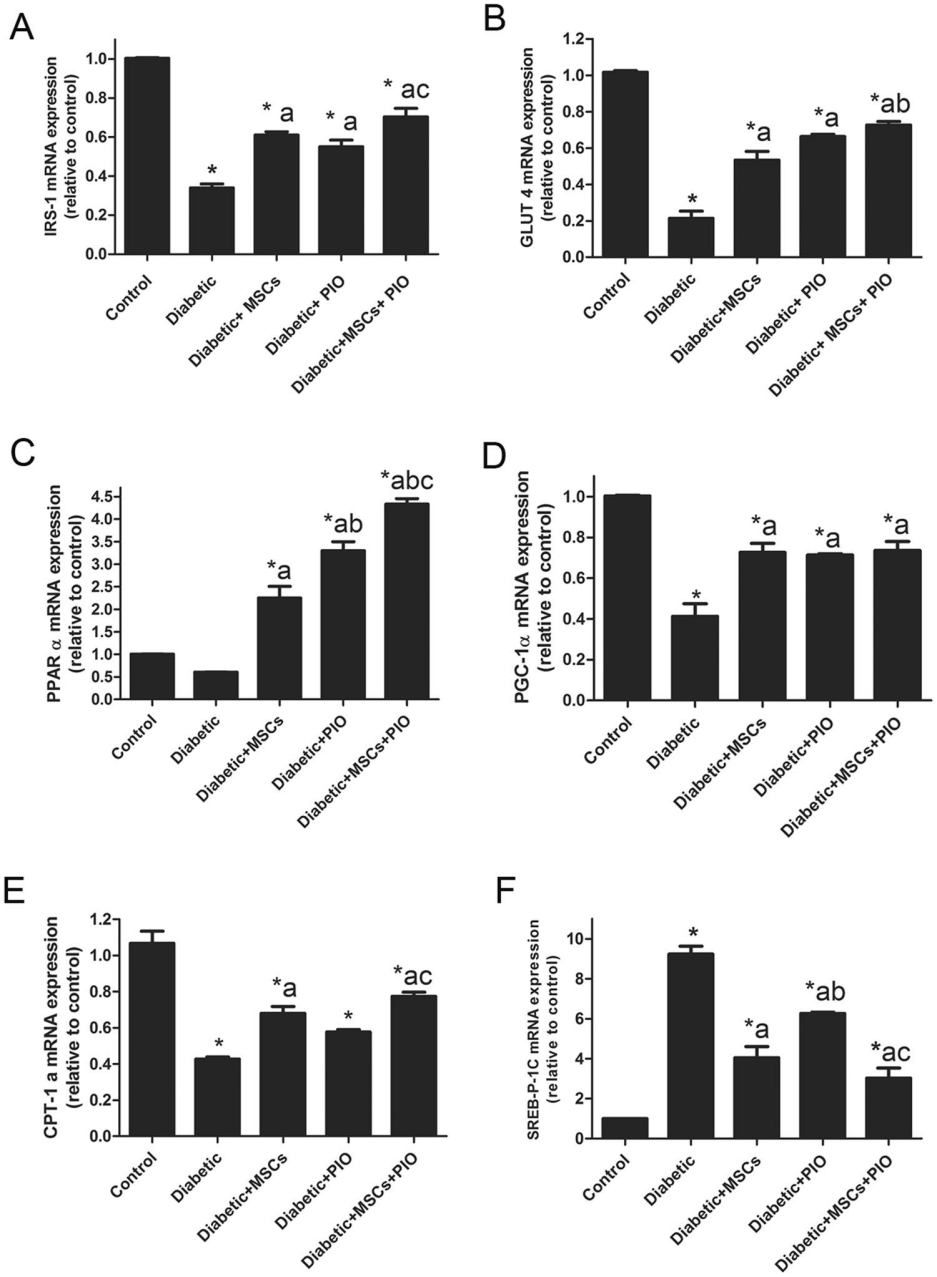
Effects of MSCs, PIO and their combination on mediators of cardiac glucose and fatty acid uptake and metabolism of diabetic rats. Cardiac mRNA levels of IRS-1, GLUT4, PPARα, PGC-1, CPT1a and SREBP-1c. All the experiments were repeated in 2 times and all data are presented as Mean ± SEM (n = 3 animals per group). *P < 0.05 vs. control; a P < 0.05 vs. diabetic group; b P < 0.05 vs. Diabetic + MSCs group; c P < 0.05 vs. Diabetic + PIO group.

Among insulin sensitivity, glucose and FA regulating genes, expressions of cardiac IRS-1, GLUT4, PPARα, PGC-1, CPT1 and SREBP-1c genes were evaluated in the present work. Cardiac mRNA levels of GLUT4 and IRS-1 (Fig. 4) were significantly decreased in diabetic rats. In contrast, cardiac mRNA levels of GLUT4 and IRS-1 were significant elevated in diabetic + MSCs, diabetic + PIO groups and their combination group as compared to diabetic group. MSCs + PIO further significantly increased heart levels of GLUT4, compared to MSCs + diabetic group.

Whereas, there are no significant difference between this group compared with Diabetic + PIO group. The present work shows a significant decrease in the mRNA expression levels of PGC-1 and CPT-1 in diabetic heart and non-significant decrease in PPARα accompanied by increased mRNA expression level of SREBP-1c. After treating diabetic rats with MScs, PIO, or their combination, the expressions of PPARα, PGC-1 and CPT-1 were obviously increased and mRNA expression level of SREBP was decreased as compared to diabetic group. Additionally, there was a significant increase in mRNA levels of IRS-1, PGC-1 and CPT-1 with a significant decrease in level of SREBP in diabetic + MSCs + PIO group when compared to PIO group indicating that the combination has more protective effects than PIO.

### Effects of MSCs, PIO and their combination on oxidative stress markers and inflammatory markers in hearts of diabetic rats

**Figure 5:**
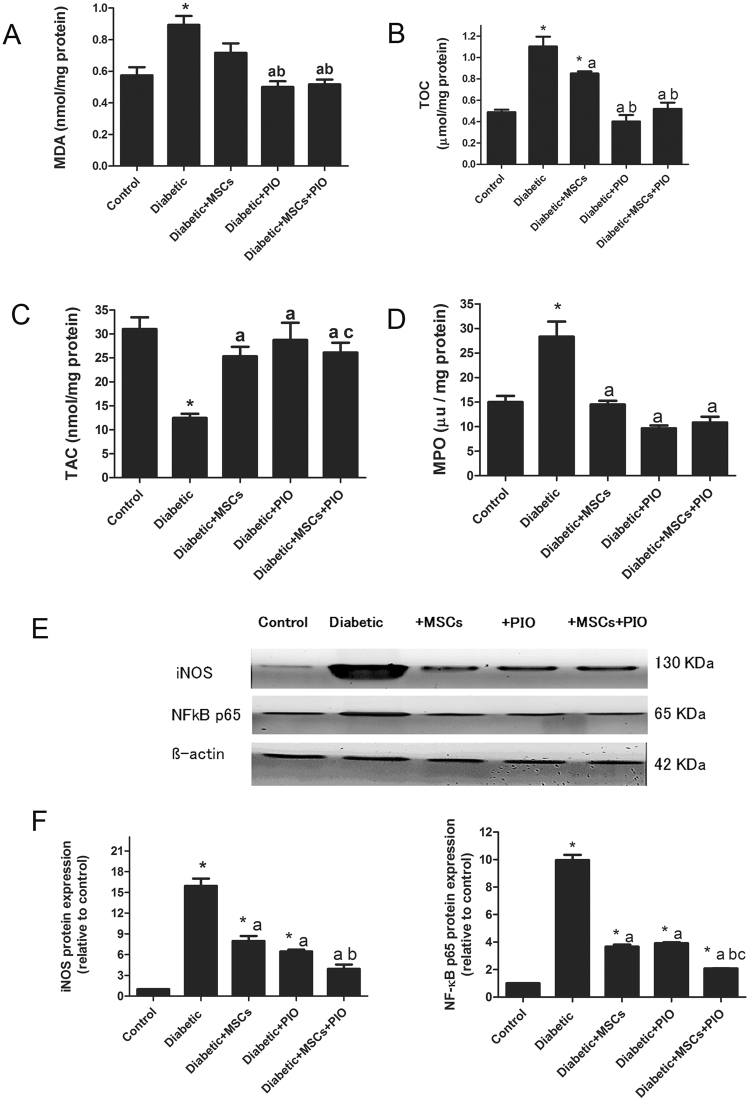
Effects of MSCs, PIO and their combination on oxidative stress and inflammatory markers in heart of diabetic rats. Cardiac MDA level (**A**), TOC (**B**), TAC (**C**), and MPO activity (**D**). All the experiments were repeated in 2 times and all data are presented as Mean ± SEM (n = 6–8 animals per group). *P < 0.05 vs. control; a P < 0.05 vs. diabetic group; b P < 0.05 vs. Diabetic + MSCs group. E & F represent Western blotting assay and quantitative analysis of iNOS and NFkB protein expressions in heart tissue of experimental animals. Expression of β actin was used to document equal protein loading. Data are expressed as fold change (relative to control group) WB was repeated in 2 times and all data are presented as Mean ± SEM (n = 3 animals per group). *P < 0.05 vs. control; a P < 0.05 vs. diabetic group; b P < 0.05 vs. Diabetic + MSCs group; c P < 0.05 vs. Diabetic + PIO group.

Cardiac MDA level, TOC and MPO activity were significantly increased and TAC was significantly decreased in hearts of diabetic rats (Fig. 5), indicating the potent oxidative action of diabetes on cardiac tissues. Cardiac MDA level and MPO activity was significant decreased and TAC was significantly increased in diabetic + MSCs group as compared to control and diabetic groups. The treatment with PIO or MSCs + PIO further suppressed heart levels of different oxidative stress markers, compared to diabetic and diabetic + MSCs groups. While there was no significant difference between diabetic + PIO or diabetic + MSCs + PIO groups.

Cardiac inflammation in the diabetic rats were evaluated by the protein expression of NF-kB and iNOS in heart tissues (and MPO activity). Compared with control group, the protein expressions of NF-kB and iNOS, were significantly elevated in diabetic group (Fig. 5E,F), indicating the potent inflammatory action in diabetic heart. After treatment with MScs or PIO, the expressions of NF-kB and iNOS were significantly decreased as compared to control and diabetic groups. Moreover, the combination treatment with MSCs and PIO for diabetic rats, further extend the significant reduction of the protein expressions of NF-kB and iNOS compared to diabetic diabetic + MSCs, diabetic + PIO groups.

### MSC and PIO treatment and their combination reduced histopathological lesions of hearts of diabetic rats

**Figure 6:**
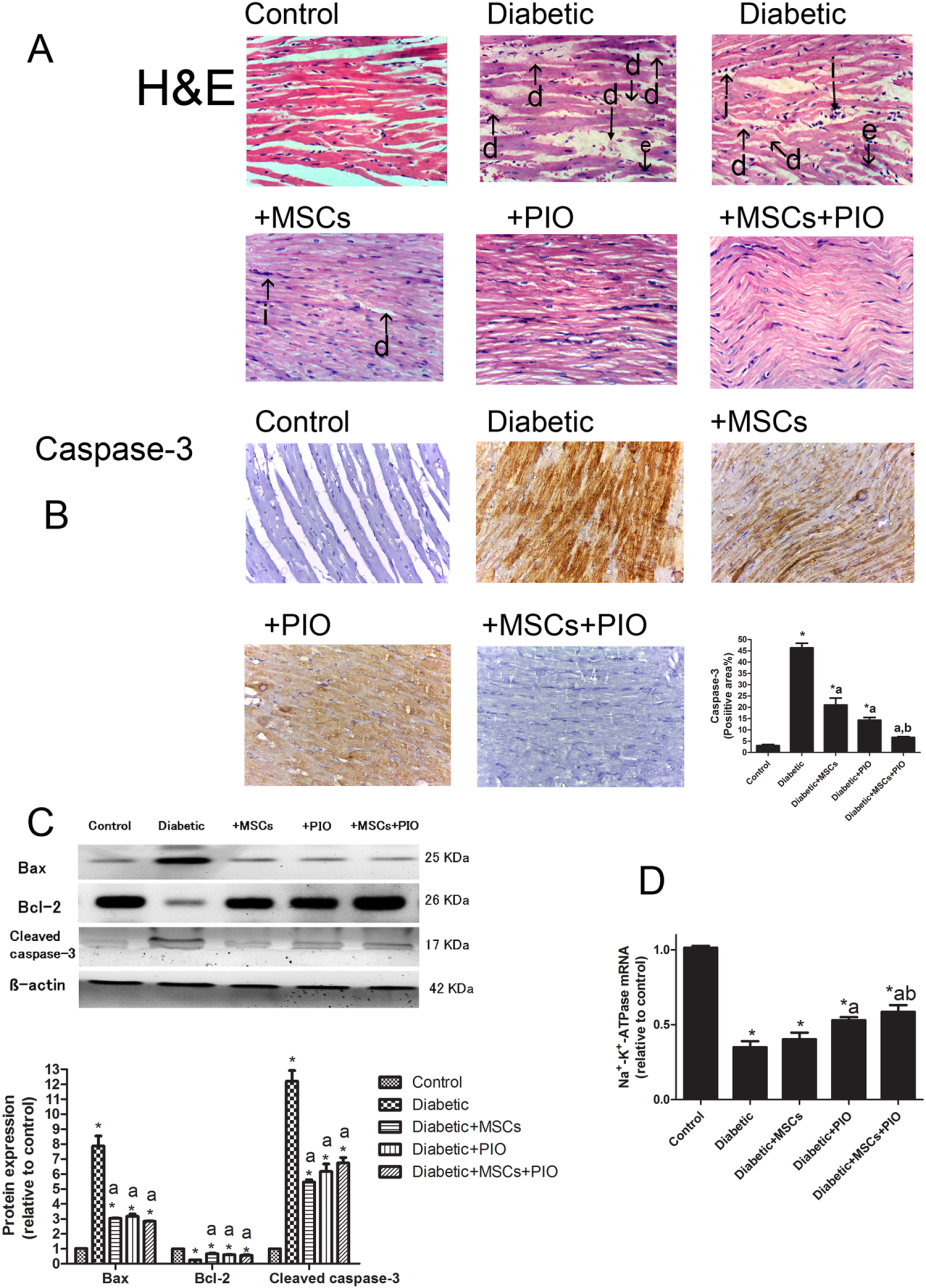
(**A**) Representative H&E-stained cardiac sections of the different groups (400x). Myocardium of rats of the control groups showing normal branching of myocardial fibers with acidophilic cytoplasm and centrally located, oval vesicular nuclei. Myocardium of diabetic rats showing inflammation (i), eosinophilic staining (e) and degeneration (d) in myocardial structure. Myocardium of rats of diabetic rats-treated with MSCs and/or PIO group showing minimal inflammation, eosinophilic staining and degenerations. (**B**) Representative IHC images showing expression of caspase 3 in the myocardium (400x). Intensity of dark brown staining indicates sites where caspase-3 level is expressed and from which the caspase 3-positive area were quantitatively analyzed. (**C**) The cardiac protein expression of Bcl-2, Bax and cleaved caspase 3 were examined by Western blotting assay. (**D**) RT-PCR analysis of the expression of Na-K-ATPase at mRNA level in each group. Data are presented as Mean ± s.e.m. *P < 0.05 vs. control group; a P < 0.05 vs. diabetic group; b P < 0.05 vs. Diabetic + MSCs group.

Next, the histopathology of the heart and caspase-3 expression were examined to determine effects of MSCs and PIO on diabetes-induced pathological damage. Normal myocardial tissue in the form of branching and anastomosing cardiac muscle fibers with acidophilic sarcoplasm and centrally located nuclei (Fig. 6A), while microscopic examination of the heart specimens of the rats of the diabetic group showed moderate degeneration of the myocardium with congestion of blood vessel (Fig. 6A and Table 1). Consistent with this pathological changes, mild inflammatory cells infiltration was found in hearts of this group of rats. As compared to diabetic group, diabetic + MSCs and Diabetic + PIO showed mild degenerative and inflammatory changes in myocardium. The histopathological changes were even less in Diabetic + MSCs + PIO group as reflected by less degeneration and inflammation as compared to the diabetic group.

**Table 1:** Scores of Histopathology in heart tissues by H&E (0–3).

	Control	Diabetic	Diabetic + MSCs	Diabetic + PIO	Diabetic + MSCs + PIO
Inflammatory cells infiltration (i)	0	2	2	1	1
Eosinophilic staining (e)	0	3	2	1	1
Degenerative changes (d)	0	3	2	1	1
Congestion of blood vessel	0	3	0	0	0

Scores represent values obtained from tissue sections of 3 animals of each group, 10 fields/section (X40). Scores 0, normal; 1 mild; 2 moderate; 3 severe levels.

Moreover, high distribution of caspase-3 was observed in the myocardium of diabetic rats (Fig. 6B). In diabetic rats which received MSCs and PIO treatment, a relatively lower caspase-3 distribution was observed in myocardium. In addition, the determination of cardiac protein expression related to apoptosis by WB revealed that the anti-apoptotic protein Bcl-2 was significantly higher, while the apoptotic proteins cleaved caspase-3 and Bax were lower in the hearts of diabetic rats when compared to normal rats. MSCs and/or PIO treatment considerably normalized the diabetic changes in the expression of these proteins. Treatment of diabetic rats with MSCs or PIO resulted in significant increase in Bcl-2 expression in the heart (P < 0.05) and significant decreased in proteins expressions of cleaved caspase-3 and Bax when compared with control and diabetic groups. Moreover, in diabetic rats treated with MSCs + PIO were significantly lower when compared to diabetic and MSCs groups. The present work shows a significant decrease in cardiac Na+-K+-ATPase mRNA expression levels in diabetic rats (Fig. 6D). Treatment of diabetic rats with PIO resulted in significant increase Na+-K+-ATPase mRNA expression levels when compared with diabetic group. Moreover, increase Na + -K + -ATPase mRNA expression were more evident in in diabetic rats treated with MSCs + PIO.

### Effects of MSCs, PIO and their combination on fibrotic markers in heart of diabetic rats

**Figure 7:**
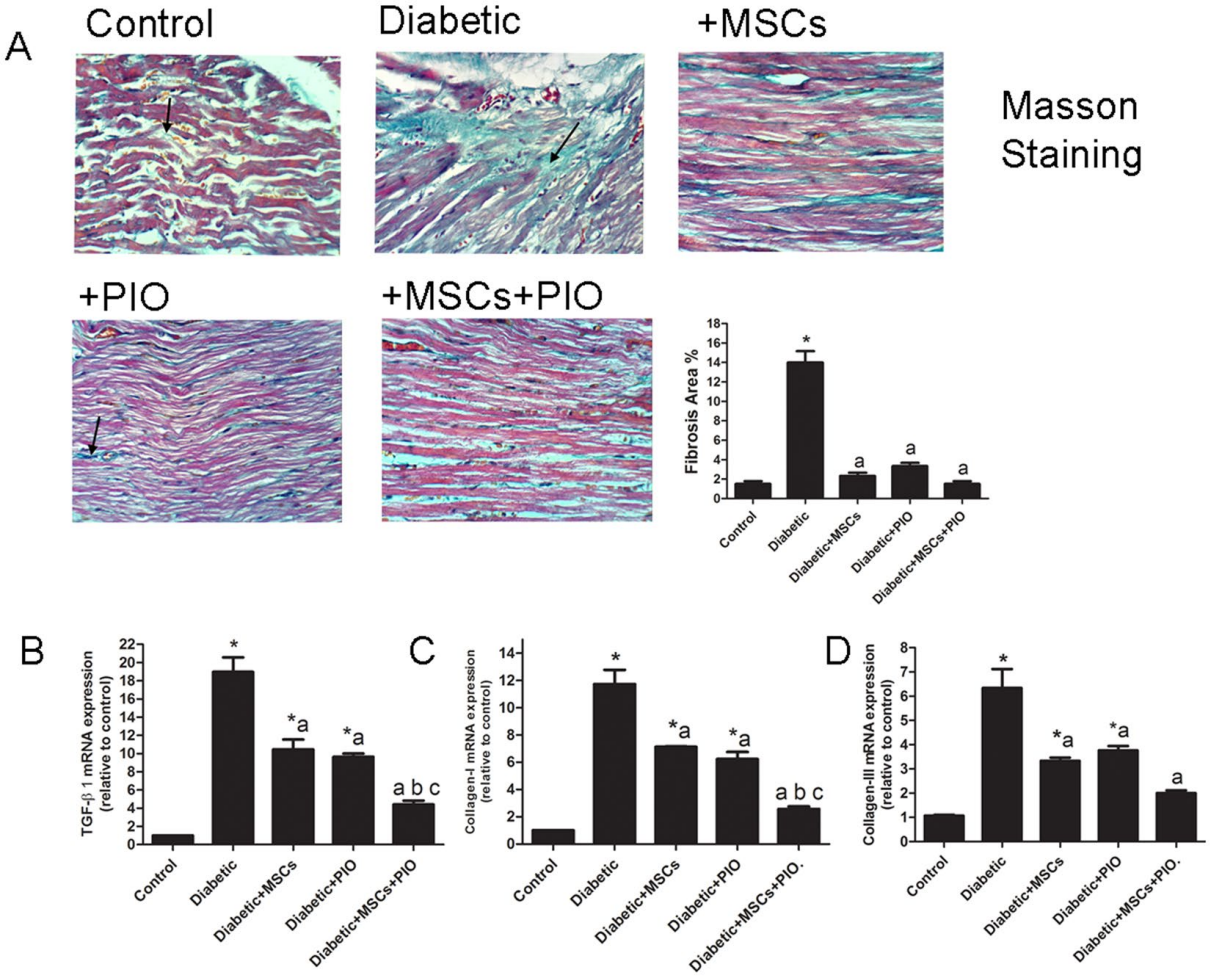
(**A**) Representative Masson trichrome-staining of cardiac sections of the different groups (400**x**). Arrows indicated intercellular collagen. Myocardium of rats of diabetic rats-treated with MSCs and/or PIO group showing minimal mason staining. (**B**) RT-PCR analysis of the expression of TGF-β, collagen I, and III at mRNA level in each group. All the experiments were repeated 3 times and all data are presented as Mean ± SEM (n = 3 animals per group). *P < 0.05 vs. control; a P < 0.05 vs. diabetic group; b P < 0.05 vs. Diabetic + MSCs group; c P < 0.05 vs. Diabetic +PIO group.

Cardiac fibrosis and accumulation of collagens in the diabetic groups was observed by Masson staining (Fig. 7A) and MSCs and/or PIO treatment significantly attenuated the cardiac fibrosis in diabetic rats. Furthermore, we examined mRNA levels of TGF-β, collagen I and III as diagnostic markers of cardiac fibrosis. Diabetic groups had significantly elevated TGF-β collagen I and III levels and the up-regulation of these fibrotic markers were remarkably inhibited in Diabetic + MSCs and Diabetic + PIO groups compared to control and Diabetic groups. Moreover, reductions of TGF-β, collagen I and III expressions were more evident in the diabetic group of rats treated with MSCs + PIO compared to Diabetic + MSCs and Diabetic + PIO groups.

### Identification of implanted MSCs in myocardium

MSCs were immunostained with PKH26 fluorescent dye to examine whether MSCs were appeared in whole diabetic pancreases and hearts. As shown in Fig. 1 supplementary, PKH26 expressing MSCs were detected in the diabetic heart at 3 weeks flowing MSCS injection. However, PKH26 fluorescent dye expressing MSCs were not detected in pancreases of diabetic-treated groups (Data not showed). Transplanted MSCs were observed in the heart of diabetic rats that received MSCs, and MSCs + PIO as indicated by PKH26 localization. These data suggest that a few MSCs were engrafted in diabetic hearts and remained there up to 3 weeks. Notably, numerous areas of PKH26-MSCs positive cells were detected in the MSCs and PIO group relative to the MSCs group. These data indicate that the *in vivo* survival of MSCs following transplantation is significantly increased by treatment with MSCs + PIO.

## Discussion

PIO is reported here and for the first time to promote the BMCs’ potential to protect against diabetes-induced cardiac damage, fibrosis, oxidative stress and inflammation. BMCs’ supporting effect of PIO was mediated by PGC-1α and PPARα pathways. Combined therapy could then improve cardiac insulin sensitivity, anti-inflammatory, anti-apoptotic, anti-fibrotic effects during the subacute period of diabetes. In the present model, hyperglycemia was associated with dyslipidemia, significant depletion of insulin, poor HOMA-β cell function and morphological damage and fibrotic pancreas. This impaired the β -cell function and led to reduced insulin production and secretion. Those defects have been well correlated with degenerated islets of Langerhans in T2DM animal model^3^. Here, MSCs-treated diabetic rats showed a transient decrease of blood glucose following MSCs infusion with no significant effect on β cell function in HOMA- β cell function. Infusion of one dose of MSCs has exhibited similar short-term effects and failed to restore normoglycemia in T2DM rats induced by high-fat diet and STZ to rats^17,20^. In other diabetic models, the administration of a single dose of adult MSCs has neither shown curative effect on pancreatic regeneration^18^ nor did it improve blood glucose^19,25^ and insulin resistance of those diabetic rats^16^.

Compared to MSCs treatment, PIO, alone, markedly improved serum glucose and insulin level and sensitivity as well as β cell function (HOMA-IR and HOMA- β cell function). Interestingly, combined treatment potentiated PIO’s protective effects. Indeed, PIO has been shown to be essential for controlling glucose homeostasis, improving insulin resistance and secretion and reducing the progression of β cell dysfunction in animal diabetic models^7,12,26,27^. The present investigation also showed that dyslipidemia, destruction of the pancreas and fibrosis were markedly reduced in diabetic rats receiving PIO. Whereas in diabetic rats receiving both MSCs infusion and PIO, the lesions and fibrosis in pancreatic tissue were prevented possibly through modulating insulin sensitivity and islet regeneration. To that end and for the first time, we report here that the protective effects of MSCs and /or PIO are associated with attenuated diabetic-induced depletion of mRNA levels of genes that modulate insulin secretion and metabolic regeneration of pancreatic cells; namely PPAR α, PGC-1α, GLP-1 and IRS-2. Increased glucose level with its reduced utilization by pancreatic cells has been reported to down-regulate PPAR α, and aggravate lipotoxicity^28^. Expression of PPAR α, PGC-1α, GLP-1R and IRS-2 genes were down regulated in this study. PGC-1α is a master regulator of mitochondrial genes where its reduced expression often relates to impaired oxidative phosphorylation in islets of T2DM patients^29^. PPARα, on the other hand, control of glucose homeostasis, FA catabolism, cell differentiation, anti-oxidative stress, and cell protection^10^^,^^28^. PGC-1 mRNA levels are significantly increased in islets from Zucker diabetic fatty rats and ob/ob mice^30^ and decreased in islets from patients with T2DM^31^. GLP-1R and IRS2 are two pancreatic factors involved in to β-cell preservation directly by enhancing β-cell proliferation and preventing apoptosis, and indirectly by lowering the toxicity levels of glucose and lipids to β-cells^32,33^. Present results showed that the protective effects of either of MSCs or PIO are concomitant with attenuated diabetic-induced depletion of mRNA levels of GLP-1R and IRS-2 in pancreatic tissue. The up regulation of expressions of all four genes explains, at least in part, the reason why MSCs and PIO restored pancreatic function. Moreover, we show that dual treatment with MSCs and PIO up regulated PPAR α, PGC-1α, GLP-1R and IRS-2 gene expressions more efficiently than either alone suggesting that the combined suppressive effect of MSCs + PIO treatment against pancreatic damage and β-cell dysfunction is mediated by improving glycemic and lipid metabolism of pancreatic tissues. GLP-1 is an important incretin hormone that improves β-cell function, stimulates insulin secretion and increases β-cell mass by enhancing β-cell proliferation^32^. It is produced locally in the pancreas and its expression and secretion within islets are regulated by various factors such as cytokines, hyperglycemia, and β-cell injury^33^. It has been reported that GLP-1 stimulates β-cell regeneration, differentiation and prevents apoptosis via the activation of GLP-1R^34^. IRS2 induction in β-cells contributes to increasing their function and mass via enhancing insulin signaling^27^. Thus, the suppressive effect of MSCs + PIO treatment against pancreatic damage and β-cell dysfunction shown here might be mediated by the improvement of glycemic and lipid metabolism of pancreatic tissues.

Impaired cardiac insulin sensitivity and metabolic dysregulation are emerging as major molecular and metabolic mechanisms of cardiac dysfunction^35^. Among the cardiac glucose and FA regulating genes are IRS-1 and IR-2 GLUT4, PPAR, PGC-1, CPT1 which were evaluated in the present study. Cardiomyocytes obtain the majority of their ATP from the oxidation of FA, high levels of plasma free FA, as occur during diabetes^36^. Glucose transporter 4 (GLUT4) is the most abundant glucose transporter isoform and primarily contributes to insulin-stimulated glucose uptake in the cardiac muscle^37–39^. The GLUT4 expression level in the myocardium were reduced in T2D animals and patients^38–40^. This reduction has been shown to elevate insulin resistance and to worsen the myocardial dysfunction^41^. Importantly, insulin receptor substrate (IRS-1) helps transmitting insulin signaling in cardiac myocytes and regulating the cardiac energy metabolism^9,42^. Insulin resistance that impairs the expression and translocation of GLUT4, and disturbs insulin signaling pathway in cardiomyocytes, was observed in various animal T2D models^43^. Consistently, the present investigation showed significant decrease in the expressions of IRS-1 and GLUT4 in the heart of T2D rats. Therefore, improving cardiac insulin sensitivity, glucose transport and metabolism are potentially excellent targets in the treatment of T2DM. Here, infusion of MSCs and/or PIO administration increased cardiac GLUT4 mRNA expression in all treated groups, thus improving the cardiac glucose metabolism. The present data demonstrated that PIO and/or MSCs improved cardiac insulin resistance through decreasing the down-regulation of cardiac IRS-1 in T2D rats. Previously, MSCs were able to restore the expression of GLUT4 protein in skeletal muscles and adipose tissues of T2D rats through an insulin-independent pathway^17^. Yang *et al*.^25^ have indicated that repeated infusion of MSCs alleviated the cardiac injury and improved heart dysfunction and elevated IR, IRS1 and p-Akt protein levels in T2D rats. Additionally, it has been reported that synthetic thiazolidinedione ligands of PPARγ and PIO improved insulin sensitivity in T2D and induce GLUT4 mRNA expression in adipose and muscle tissues^37,40^. The metabolic readout of the T2D heart and its associated insulin-resistance is characterized by excessive FA utilization and storage^9,35,42,44^. During diabetes, and owing to the insulin resistance and the dysregulation of glucose utilization, diabetic heart relies almost exclusively on FA oxidation as the main ATP source^36,45^. Among other FA regulating genes studied here, PPARα is known to be predominantly expressed in heart with an elevated capacity for FA oxidation, and its activation increases FA uptake and subsequent import into mitochondria for oxidation^9^. PPARα-induced FA uptake is often mediated by modulating the expression of carnitine palmitoyltransferase 1 (CPT-1) activity which acts as a rate-limiting enzyme of mitochondrial FA oxidation^42^. For mitochondrial import, the fatty acyl-CoAs are transferred to CPT1 generating acylcarnitine^36^. PGC-1α is the master regulator of cardiac mitochondrial biogenesis^36,46^. At the transcriptional level, PGC-1α co-activates PPARα to regulate the expression of genes involved in the electron transport chain, mitochondrial biogenesis, and FA β-oxidation and glucose oxidative metabolism^45^. The present work reported a significant decrease in the gene expression of PGC-1, PPARα and CPT1in diabetic heart accompanied by increased mRNA expression of the lipogenic gene; SREBP-1c. This is in agreement with results from both diabetic animal models and human subjects where FA oxidation is significantly reduced during cardiovascular abnormalities^45^. Reduced expressions of target genes (PPAR α and PGC-1α) which are known to be associated with impaired energy metabolism, were observed during cardiac dysfunction in diabetes^46^ and in heart of diabetic animal^47^. In fact, during the late stages of diabetic heart, the activity and expression of PGC- 1α are decreased, leading mitochondrial dysfunction and cardiac hypertrophy and dysfunction^24,48^. Recent studies have shown that mitochondrial function is altered in the hearts of type one diabetic animals^24^ and in T2D model^47,49^. Yan *et al*.^49^ demonstrated that impairment of downstream AMPK-PGC-1α signaling directly contributed to reduced mitochondrial biogenesis and function in ob/ob mouse hearts. Treatment with PIO increased expression of PGC-1α and of genes in the FA oxidation pathway including PPAR-α, CPT-1 in adipose tissue of T2DM patients^50^. MSCs infusion to infracted heart of rats conserved glucose uptake in the heart. This effect was linked to the ability of the MSCs treatment to overcome the decline in insulin signaling, IRS-1 and to prevent alterations in PGC-1 and its downstream signaling^51^. MSCs-conditioned medium was also shown to ameliorate vascular injury in diabetic rats via regulating reactive oxygen species (ROS) levels and mitochondrial function in endothelial cells and activation AMPK/PGC-1α pathway^52^. The MSCs/PIO-induced recovery of GULT4, PPAR and SREBP expression levels shown here suggested a potential of such dual therapy to improve in cardiac biogenesis and decrease accumulation of gluco/lipotoxicity. Targeting metabolic derangements via improving glucose oxidation and/or FA oxidation has been reported as a promising therapeutic strategy to treat diabetic heart failure^10^.

Notably, the activation of PPAR/PGC-1 α signaling not only could prevent glucose and lipid metabolic disturbance but could also inhibit inflammatory process in diabetic heart. Diabetes is closely associated with chronic inflammation^6,42^. The progression of diabetic cardiomyopathy usually entails a local rise in various molecules related to the inflammation such NF-κB. Diabetes activates the NF-kB signaling, which regulates the expression of several genes involved in the inflammatory response and cellular stress including iNOS^53,54^. Levels of inflammation in heart of diabetic rats were high as evidenced from the elevated levels of NF-κβ and iNOS^55,56^. NF-κB is activated by hyperglycemia or free radical linking oxidative damage and inflammation where increased expression of iNOS catalyzed the production of large amount of NO^9^. Increased oxidative stress in the diabetic heart is a contributing factor in the development of diabetic cardiomyopathy^6,57^. Consisting with previous reports^56,58^, diabetic heart damage and apoptosis shown here was associated with enhanced MDA and TOC as well as depletion of TAC and treatment with MSCs reduced the levels of oxidative stress in heart of diabetic rats (lower MDA level and TOC and higher TAC) when compared to diabetic control rats. In this study, diabetes caused elevations of cardiac MPO activity, a marker of neutrophil infiltration and inflammation indicating the presence of enhanced neutrophil recruitment in the inflamed tissues. In MSCs or PIO-treated diabetic group, cardiac MPO activity was attenuated indicating the anti-inflammatory action of MSCs or PIO. MSCs treatment has also been shown to ameliorate diabetic nephropathy by secreting hepatocyte growth factor (HGF) which in turn reduced macrophages infiltration, down-regulated IL-1β, IL-6, TNFα expression in renal tissue in STZ diabetic rats^59^. MSCs have been reported to uniquely possess a differentiation promoting capacity for tissue repair and the aptitude to modulate the immune system and thus controlling inflammation^14^. Similarly, thiazolidinedione treatment has been shown to reduce both lipid radical formation and hepatic iNOS protein levels suggesting a potential mechanistic relation between these processes where decreased oxidative stress was also reported to be associated with better metabolic response and insulin signaling^60^. Kadiiska *et al*.^60^ suggested that thiazolidinedione treatment can reduce oxidative stress in insulin resistance rat model through reducing iNOS-derived lipid radical formation. Previously, PIO attenuated macrophage infiltration into the myocardium as well as the up-regulation of pro inflammatory markers such as TNF-α, in the heart of DS/obese rats^13^. In addition, PIO ameliorated cardiac oxidative stress in DS/obese rats^13^. Interesting, an improvement in oxidative stress and inflammatory status was observed here when MSCs was combined with PIO as evidenced by a decrease in cardiac MDA and NFkB compared to MSCs and PIO treated groups and that correlated well with upregulation of PPARα. In fact, several investigations have also shown that the activation of PPARs exerts cardio protective effects by inhibiting NF-kB signaling pathway, thereby suppressing inflammatory responses^9,61^. PPAR activation is capable of limiting myocardial inflammation through several mechanisms, such as the physical interaction between PPARs and the p65 subunit of NF-κB, or, the induction of anti-oxidative genes, the release of nuclear co-repressor, or the inhibition of MAPK phosphorylation^9^.

Diabetes-induced heart damage, apoptosis and fibrosis are the most prominent feature in diabetic animals^55,56,62,63^. In this study, histopathological changes indicating of tissue injury were noted in diabetic rat heart including degeneration, inflammation, positive expression of caspase-3, induced level of cardiac pro-apoptotic markers (cleaved caspase-3 and Bax) and the inhibition of anti-apoptotic marker (Bcl-2) in the heart of diabetic rats. MSCs and PIO treatment protected the rats against diabetic induced heart injury and apoptosis. These cardiac pathological abnormalities were remarkably ameliorated by treatment with either MSCs or PIO in diabetic rats. Moreover, the combination of MSCs and PIO potentiated the decrease in cardiac injury in diabetes and these abnormal myocardium pathological findings were significantly reduced in this group compared to MSCs group and PIO group. Furthermore, the combined therapy applied here of MSCs and PIO reduced apoptosis in the heart in diabetes as evident by lowering levels of pro-apoptotic markers i.e. caspase-3, cleaved caspase-3 and Bax and increasing the levels of anti-apoptotic marker, Bcl-2 in the heart of diabetic rats. MSCs and adipose-derived MSCs transplantation was reported to reduce STZ-induced renal injury and renal tissue cell apoptosis^64^, and to decrease the apoptosis rate and the expression of Bax and elevated Bcl-2 in kidney in diabetic rats^65^. Umbilical cord blood –MSCs, have also been shown to improve cardiac structure and function in a transgenic mouse of dilated cardiomyopathy mouse, an effect that was associated with reductions in cellular apoptosis, inflammation, hypertrophy and myocardial fibrosis and up-regulation of Bcl 2^21^. Na+/K+-ATPase is a classical membrane-bound pump that exchanges Na+ and K+ across cell membrane for maintaining electrochemical gradient and cell membrane potential. Recent study showed that reduced Na+/K+-ATPase activity has a close relationship with diabetes-related cardiomyocyte death and cardiac dysfunction^66^. In line with Yan *et al*.’s findings, PIO/MSCs and their combination applied here did alleviate the decrease in cardiac Na+-K+-ATPase mRNA expression levels in diabetic rats.

Cardiac fibrosis is an important contributor to the pathogenesis of heart failure both in type 1 and type 2 diabetes and activation of TGF-β 1 signaling may activate fibroblasts inducing deposition of structural extracellular matrix proteins such as collagen, which increases myocardial stiffness^67^. In the model of diabetes presented here, the pathological characteristic of diabetic heart displayed an accumulation of intracellular fibrosis, significant elevation of mRNA levels of TGF-β 1, collagen-I and collagen-III. Cardiac fibrosis has been documented in experimental models of type 1 and type 2 diabetes^62,68,69^. Present results demonstrated that both MSCs and PIO treatment significantly attenuated the increased cardiac fibrosis and inhibited the mRNA level expressions of collagens and TGF-β 1in diabetic groups. These finding are consistent with the previous study where either PIO or MSCs treatments inhibited fibrosis and the up-regulation of collagen-I and collagen-III and TGF-β 1 expressions in the heart of diabetic/obese rats^13^ and in kidney of diabetic rats^64,70^. In previous *in vitro* study, MSC-CM inhibited upregulation of TGF- β expression stimulated by high glucose and secreted a significant amount of HGF which inhibited TGF-β production and antagonize the effect of TGF- β in hyperglycemic environment^64^. Interestingly, the inhibitory effects of each of MSCs on diabetic cardiac fibrosis and upregulations of TGF-β, collagen I and III gene expressions are partial but additive when combined with PIO, suggesting diverse transduction pathways are potentially involved in attenuation of cardiac fibrosis in diabetic rats including anti-oxidative, anti-inflammatory and ant-hypoglycemic properties^42,71^. PIO and MSCs have been shown to inhibit fibrosis and PPARs have already been considered an attractive therapeutic target for the treatment of metabolic disorders and PPARγ agonists were shown to effectively attenuate oxidative stress, inflammation and apoptosis^6^. In the present investigation, MSCs labeled with PKH26 fluorescent dye were detected in the cardiac tissues confirming that those cells were homed into the cardiac tissue suggesting that BMCs can reside into injured myocardium, differentiate into cardiac cells, and participate in regeneration of the highly specialized heart tissue. Other studies have shown the distribution of MSCs to the kidney^64^ and the heart^21^ of diabetic rats. Because the PKH26-labelled MSCs were not detected in the host pancreas, it is conceivable that the mechanisms that mediate the repairing capacity of MSC in diabetic pancreas are primarily paracrine rather than the direct interaction. To that end, MSCs are known to secrete numerous growth factors and cytokines^14^.

Collectively, we propose here that the potentiation of the MSCs protection against diabetic-induced heart injury, apoptosis and fibrosis by PIO treatment contributed, at least in part, to the improvement of cardiac insulin sensitivity, anti-inflammatory and anti-oxidative effects.

## Materials and Methods

### Preparation of BM-derived MSCs from rats

Under anesthesia with ether, bone marrow was harvested by flushing the tibiae and femurs of 6 weeks old male Wistar rats with phosphate-buffered saline (PBS). Nucleated cells were isolated with a density gradient (Ficoll/Paque (Pharmacia) and the resuspended cells were cultured in low-glucose Dulbecco’s modified Eagle’s medium (DMEM) with 10% fetal bovine serum and supplemented with 1% penicillin–streptomycin (GIBCO/BRL). Cells were incubated at 37 °C in 5% humidified CO_2_ for 12–14 days as primary culture or upon formation of large colonies. Upon the formation of large colonies (80–90% confluence), cultures were washed twice with PBS and cells were trypsinized with 0.25% trypsin in 1 mM EDTA (GIBCO/BRL) for 5 min at 37 °C. After centrifugation, cells were re-suspended in serum-supplemented medium and incubated in 50 cm^2^ culture flasks (Falcon). The resulting cultures were referred to as first-passage cultures and were further propagated until passage three.

### Characterization of rat BM-derived MSCs

MSCs are defined by an array of positive and negative markers and are normally plastic-adherent under standard culture conditions. They express CD105, CD73, CD90 and CD 29, but, not CD34, CD14, and CD11b^72^. In the present work, BM-derived MSCS were characterized by their adhesiveness and spindle shape and by flow cytometric evaluation; MSCs expressed CD90 and CD29 but not CD34 on their cell surface. Cells at passage 3 were analyzed by fluorescence-activated cell sorting (FACS) (Becton Dickinson, Franklin Lakes, NJ, USA). After blocking for nonspecific binding with buffer containing 1% bovine serum albumin, cells were incubated for 20 min at 4 °C with the antibodies FITC-conjugated anti-CD29, FITC-conjugated anti-CD90 and FITC-conjugated anti-CD34 (Biolegend, San Diego, CA, USA).

### Labeling of MSCs with PKH26

MSCs were harvested during the 3^th^ passage and were labeled with PKH26 (Sigma, St. Louis, MO, USA), which is a red fluorochrome. Cells were centrifuged and washed twice in serum free medium. Cells were pelleted and suspended in dye solution. Labeled cells retained both biological and proliferating activity, and were ideal for *in vitro* cell labeling and proliferation studies and for extended *in vivo* cell tracking. In the current work, cells were injected intravenously (2 × 10^6^ MSCs suspended in 0.2 mL normal saline) into tail vein at 7 days after T2DM induction^17^. After one month of T2DM, heart and pancreas tissue was examined with fluorescence microscope to detect and trace the cells. The fluorochrome has excitation (551 nm) and emission (567 nm) characteristics compatible with rhodamine or phycoerythrin detection Systems.

### Animals and experimental design

Male, six-weeks-old adult Wistar rats, weighing approximate 120–150 g were received from the animal house of the National Organization for Drug Control and Research (NODCAR). They were maintained on standard pellet diet and tap water ad libitum and were kept in polycarbonate clean cages under a 12 h light/dark cycle and room temperature 22–24 °C. Rats were acclimatized for 2 wks. prior to experimental use. Animals were cared for in accordance with the standard guidelines (Canadian Council on Animal Care 1993) were acclimatized for 2 wks. Prior to experimental use. The protocol was approved by NODCAR Ethics Committee of Animal Care and Use. All experiments were performed in accordance with relevant guidelines and regulations.

### Induction of rat T2DM model

Induction of T2DM was done using a combination of intraperitoneal injections of streptozotocin (STZ) dissolved in 0.1 M sodium citrate buffer, pH 4.5, and of nicotinamide (NA) dissolved in normal saline according to the dosage reported in previous studies^73,74^. Briefly, rats were injected intraperitoneally with 150 mg/kg of NA 15 minutes prior to STZ injection (65 mg/kg). The animals of T2DM groups were allowed to drink 5% glucose solution for 12 h to avoid hypoglycemia. After 3 days of STZ injection, the fasting blood glucose level from the tail was determined to ensure successful induction of hyperglycemia by using a commercial glucometer (Accu-Check glucometer, Hoffman-La Roche Ltd., Basel, Switzerland). Rats having blood glucose above 250 mg/dL were considered diabetic and were utilized for the experiments. Diabetes was induced in 32 rats of 40 rats, as described previously. One week after the verification of diabetes, T2DM rats were further randomly divided into four groups consisting of eight animals in each group (n = 8). The combined effect of STZ and NA in rodents was reported to produce T2DM^73,74^. STZ has been shown to decrease nicotinamide adenine dinucleotide^72^ levels in the pancreatic β cells that, in turn, induce intracellular free radicals that ultimately result in their necrosis and/or apoptosis^74^. Administration of STZ and NA to adult rats has been reported to cause partial destruction of β-cells leading to a decrease in blood insulin and an increase in blood glucose in these animals. Rats with STZ– NA-induced diabetes have also been characterized by moderately decreased β-cell mass, pancreatic β-cell dysfunction and impairment in glucose-stimulated insulin secretion. This model has been shown to be useful particularly when diabetic complications such as cardiovascular defects and the anti-diabetic properties of new drugs are investigated^75^.

### Treatment Conditions

Forty rats were randomly divided into five groups (n = 8), the first group, the control group, was injected with single dose of citrate buffer (5 ml/kg b.wt.) and received 5 ml/kg vehicle of PIO through oral gavage for 4 weeks. The second group, the diabetic treated with vehicle. The third group was 1-week diabetic rats treated with MSCs (2 × 106 MSCs suspended in 0.2 mL saline; Diabetic + MSCs), the fourth group was 1-week diabetic rats treated with PIO (EGPI, Egyptian Group Pharmaceutical Industries) (Diabetic + PIO), and the fifth group was 1-week diabetic rats treated with MSC and PIO (Diabetic + MSCs + PIO). A suspension of PIO in 5 ml/kg/day of distilled water and tween 80 was administered to both of Diabetic + PIO and Diabetic + MSCs + PIO groups by oral gavage at a dose of 20 mg/kg b.wt. daily for 4 weeks. This PIO dose was selected in the range that induced cardio protective effect and decreased insulin resistance in different rodent models of diabetes^13,76^. Meanwhile, rats of the control and Diabetic groups only received 5 ml/kg/day of vehicle through oral gavage.

### Sample preparation

Toward the end of the treatment period, blood glucose levels were determined utilizing glucometer, and afterward the rats were anesthetized with diethyl ether. The Blood was collected from the retro-orbital plexus and the serum was promptly isolated by centrifugation in a refrigerated centrifuge (4 °C) at 3000 r.p.m. for 20 minutes and the collected sera were stored at −80 °C until examination. The rats were euthanized by cervical dislocation under diethyl ether anesthesia. Heart and pancreas were removed, washed with ice-cold normal saline solution and dried on filter paper. The hearts were removed, and weighed to calculate the heart to the body weight ratio (HW/BW). Parts of pancreas and left ventricles was fixed in 10% buffered formalin for histological and immunohistochemical analyses. The remaining pancreas and left ventricles were immediately snap-frozen in liquid nitrogen and stored at −80 °C for PCR and biochemical, and western blot analyses. The left ventricle was removed and sectioned into slices and were used for RT-PCR and WB. For biochemical analysis, left ventricle of heart was homogenized in ice-cold KCl (150 mM). The ratio of tissue weight to homogenization buffer was 1:10. Then, suitable dilutions from that were prepared to determine the levels of oxidative stress biomarkers.

### Biochemical analysis

Serum insulin levels were determined by using enzyme-linked immunosorbent assay (ELISA) kit (EIA-2048, 96 wells, DRG Instruments GmbH, Marburg, Germany) according to the manufacturer’s protocols. Fasting triglyceride^77^, total cholesterol^78^ and fasting serum glucose were measured enzymatically using Kit (Bio diagnostic, Egypt). The homeostasis model assessment of insulin resistance (HOMA-IR) and homeostasis model assessment of β-cell function (HOMA-β) indices, which predict insulin sensitivity and the function of pancreatic β-cells, respectively, were calculated from the serum glucose and serum insulin concentrations according to the empirical formulae: HOMA-IR = fasting insulin (μU/mL) × fasting glucose (mmol/L)/22.5; HOMA-β = (fasting serum insulin [μU/mL] × 360)/(fasting serum glucose [mg/dL] − 63)^79^.

### Oxidative stress biomarkers

To further, examine the effects of the treatment with MSCs and/or PIO on oxidative stress markers in heart of diabetic rats, all the following were assessed in homogenates of heart tissues. The total oxidant content (TOC) of serum samples was determined as previously described in^80^. The total antioxidants capacity (TAC) in heart was determined as reported in^81^. This method measures the change in absorbance at 593 nm due to the formation of a blue colored ferrous- tripyridyltriazine complex from colorless oxidized ferric form by the action of electron donating antioxidants. Lipid peroxidation was determined by estimating the level of malondialdehyde (MDA) as previously described in^82^. Myeloperoxidase (MPO) activity in cardiac homogenate was determined following previously published protocol^83^. The total protein content of heart was determined according to the Lowry method as modified by Peterson^84^. Absorbance was recorded using a PerkinElmer, Lambda 25 UV/VIS spectrophotometer for all measurements.

### Real-time quantitative reverse-transcriptase polymerase chain reaction (RT-PCR) analysis

Total RNA was isolated from pancreas and liver tissues of three rats of each group using SV Total RNA Isolation System (Promega, Madison, WI, USA) according to manufacturer’s instruction and was further analyzed for quantity and quality with Beckman dual spectrophotometer (USA) at 260 and 280 nm. RNA (5 µg) was then reversed transcribed and the complementary DNA (cDNA) was synthesized using Super cDNA synthesis kit Superscript III First-Strand Synthesis System as described in the manufacturer’s protocol (#K1621, Ferments, Waltham, MA, USA). For real time polymerase chain reaction (real time-PCR), the cDNA (5 µL) was subsequently amplified with the Syber Green PCR Master Kit (Applied Biosystems, Foster City, CA, USA) in a 48-well plate using the Step One instrument (Applied Biosystems, Foster City, California, USA). The PCR primers used were designed with Gene Runner Software (Hastings Software Inc., Hastings, NY, USA) from RNA sequences in Gen Bank and were represented as shown in Table 2: PCR samples were first denatured at 95 °C for 5 min followed 35 cycles. Each cycle comprised a melting step at 95 °C for 30 s, annealing steps at 60 °C for 30 s, and an extension steps at 72 °C for 30 s. The value of the cycle threshold was used to perform calculations by using the ABI Prism 7500 sequence detection system software as described previously^77^. Gene expression of each sample was analyzed in triplicates and normalized against the internal control, β-actin gene. Final results were determined as follows: 2- Δ (Ct sample- Ct control), where Δ Ct values of the control and sample were determined by subtracting the Ct value of the target gene from the value of the housekeeping gene: β-actin^77^. Data was represented as ratio or folds change to control sample.

**Table 2:** Gene-Specific Primers Used for RT-PCR.

Gene symbol	Gene ID	Primer sequence 5–3
PGC-1α	NM_008904	F 5′-AAACTTGCTAGCGGTCCTCA-3′ R 5′-TGGCTGGTGCCAGTAAGAG-3′
CPT1a	NM_013495	F 5′-TGGTCAACAGCAACTACTACGC-3′ R 5′-GAAGACGAATGGGTTTGAGTTC-3′
PPARα	NM_011144	F 5′-CAACGGCGTCGAAGACAAA-3′ R 5′-TGACGGTCT CCACGGACAT-3′
GLP-1R	NM_021332.2	F 5′-ACTTTCTTTCTCCGCCTTGGT-3′ R 5′-CCTGGTGCAGTGCAAGTGTCT-3′
SREBP-1c	NM_009204.2	F 5′-CTGGCACTAAGTGCCCTCAAC-3′ R 5′-GCCACATAGATCTCTGCCAGTGT-3′
GLUT4	NM_009204.2	F 5′-TCGTCATTGGCATTCTGGTTG-3′ R 5′-AGCTCGTTCTACTAAGAGCAC-3′
IRS-1	NM_010570	F 5′-GCCAATCTTCATCCAGTTGC-3′ R 5′-CATCGTGAAGAAGGCATAGG-3′
IRS-2	NM_001081212	F 5′-CATCGACTTCCTGTCCCATCA-3′ R 5′-CCCATCCTCAAGGTCAAAGG-3′
Na+-K+-ATPase	NM_001160234.1	F 5′-CCTGGGAGGCTTCTTCACTT -3′ R 5′-CTGCTCGTAGGTCCACTGCT-3′
Collagen I	NM_007742	F 5′-CAACCTGGACGCCATCAAG -3′ R 5′-CAGACGGCTGAGTAGGGAACA-3′
Collagen III	NM_009930	F 5′-TTGATGTGCAGCTGGCATTC-3′ R 5′-GCCACTGGCCTGATCCATAT-3′
TGFβ1	NM_011577	F 5′-ATGCTAAAGAGGTCACCC-3′ R 5′-CAAAAGACAGCCACTCAG-3′
*β*Actin	NM_031144.3	F 5′-TATCCTGGCCTCACTGTCCA-3 R 5′-AACGCAGCTCAGTAACAGTC-3

### Western blotting analysis

Heart tissue samples (50 mg) were homogenized in cold radio-immuno-precipitation assay (RIPA) solution supplemented with inhibitors for proteases and phosphatases. Protein concentrations were then determined using the established Bradford dye-binding method (Bio-Rad, Hercules, CA, USA). For direct immunoblotting V3 Western Workflow™ complete system (Bio-Rad® Hercules, CA, USA) was used, aliquots of lysate were mixed with loading buffer containing 2-mercaptoethanol and maintained at 100 °C for 10 min before loading on 10% SDS-PAGE. Following SDS-PAGE separation, proteins were transferred to PVDF membrane. Membranes were blocked in TBST containing 5% (w/v) non-fat milk and dried for 1 h at room temperature. The blot separation was visualized and imaged immediately using stain-free blot technology and ChemiDoc TM imager. Membrane strips were incubated with primary antibodies, the primary antibodies used at 1:1000 dilution included anti-NFκB-p65 (sc-372), anti-iNOS (sc-7271), anti–BCl-2 (sc-56015), anti-Bax (cs-7480), and anti-β-actin (sc-7210), all were purchased from Santa Cruz Biotechnology, Inc., Santa Cruz, CA), except rabbit monoclonal cleaved caspase-3 (9661) was purchased from Cell Signaling Technology (Beverly, MA, USA). The membranes were washed in TBST (50 mmol/L Tris–HCl, pH 7.6, 150 mmol/L NaCl, 0.1% Tween 20) for 30 min and incubated with appropriate HRP-conjugated secondary antibody (1:2,000 dilution) for 2 h at room temperature and developed by the HRP substrate 3,3′-diaminobenzidine tetrahydrochloride^72^ system (Bangalore Genei, India). Protein bands were detected by a standard enhanced chemiluminescence method and densitometry measurements were made using the ChemiDoc MP imager. The densities of target protein bands were normalized to the corresponding density of β-actin band. All signals were expressed relatively to the average values for the control group, which was set to 1.

### Histopathological examination

Pieces of pancreas and hearts were fixed in 10% neutral phosphate buffered formalin and hydrated tissue sections, 4 μm in thickness, stained with Hematoxylin and Eosin (H&E) for the histological examinations. The sections were examined under an Olympus DX41 light microscope (Olympus CX31, Honduras St., London, United Kingdom). Ten randomly chosen areas per slide were investigated from three randomly chosen animals of each group.

The sections of pancreas were graded for average severity of eosinophilic staining, degenerations, inflammations and congestion of blood vessels as follows: 0, no change; 1, mild; 2, moderate; and 3, severe^85^ as following: 1, 0 ± 10% of total myocardium; 2, 10 ± 30 total myocardium; 3, more than 30% total myocardium.

### Masson staining

Briefly, 4 μm thick paraffin-embedded cardiac and pancreatic tissue sections were stained according to standard Masson trichrome staining. Digital pictures were taken with identical exposure settings for all sections. Then, positive areas were quantified in ten randomly selected fields (magnification 400x) per individual samples. Area was quantified by use of computer-assisted image analysis software (1.39, NIH-Bethesda, MD, USA).

### Immunohistochemical analysis for caspase-3

Heart tissue sections (4 µm thick) were deparaffinized with xylene and then antigens were unmasked by immersing the sections in 0.1 M sodium citrate buffer (pH 6) in heated water bath for 15 min, followed by endogenous peroxidase blocking in 3% H_2_O_2_ for 10 min to block the endogenous peroxidase activity. After cooling, nonspecific binding was blocked by incubating with non-fat dried milk. The primary antibodies caspase 3 (sc-65497) was obtained from Santa Cruz Biotechnology, Inc., (Santa Cruz, CA). The sections were incubated with primary antibodies overnight at 4 °C. After washing the slides with PBS, then sections were incubated with polyvalent biotinylated goat anti-rabbit secondary antibody diluted 1: 200 at room temperature for 10 min. After a standard staining protocol using Universal LSAB plus kit and a DAB plus substrate kit as the chromogen, sections were counter-stained with hematoxylin. Tissue images were captured by optical microscopy (Olympus DP71). Then, intensity of dark-brown staining was quantified in ten randomly selected fields (magnification 400x) per individual samples by using ImageJ software (1.39, NIH-Bethesda, MD, USA).

### Statistical analysis

All data were statistical analysis by SPSS (version 20) statistical program (SPSS Inc., Chicago, IL, USA). The data were expressed as means ± SEM. Statistical significance between treatment groups was carry out by using one-way analysis of variance (ANOVA) followed by Tukey’s post-hoc analysis test for multiple comparisons with P < 0.05 being considered as statistically significant. Figures were done using GraphPad Prism program (version 5) (San Diego, California, USA).

## Electronic supplementary material

Supplementary information
