# Supplementary material for: Mechanistic insights into the augmented effect of bone marrow mesenchymal stem cells and thiazolidinediones in streptozotocin-nicotinamide induced diabetic rats
Source: Sci Rep. 2018 Jun 29;8:9827. doi: 10.1038/s41598-018-28029-1 (PMC6026169; doi:10.1038/s41598-018-28029-1)
Supplement: Supplementary file 1 — Supplementary information [file 41598_2018_28029_MOESM1_ESM.pdf]

Supplementary Information

Mechanistic insights into the augmented effect of bone marrow mesenchymal stem cells and thiazolidinediones in streptozotocin-nicotinamide induced diabetic rats

Alaaeldin Ahmed Hamza[1,*], Ebtehal Mohammad Fikry [2], Wedad Abdallah[3], Amr Amin[4,5,†]

1 Hormone Evaluation Department, National Organization for Drug Control and Research (NODCAR), Giza, Egypt

2,3 Department of Pharmacology, (NODCAR), Giza, Egypt

3 Department of Biochemistry, Faculty of Pharmacy, MTI University, Cairo, Egypt

4 Biology Department, College of Science, UAE University, Al-Ain, UAE

5 Department of Zoology, Faculty of Science, Cairo University, Giza, Egypt

**corresponding to: Alaaeldin A .Hamza*

E-mail:alaa17mm@gmail.com

NODCAR, 6 Abu Hazem St., Pyramids, Giza, Egypt-

Tel. 20.01201991433- Fax: +20.2.33933897

† corresponding to: Amr Amin

E-Mail: a.amin@uaeu.ac.ae

Biology Department, UAE University, UAE

Tel .97137136519 – Fax: 9713-7134927

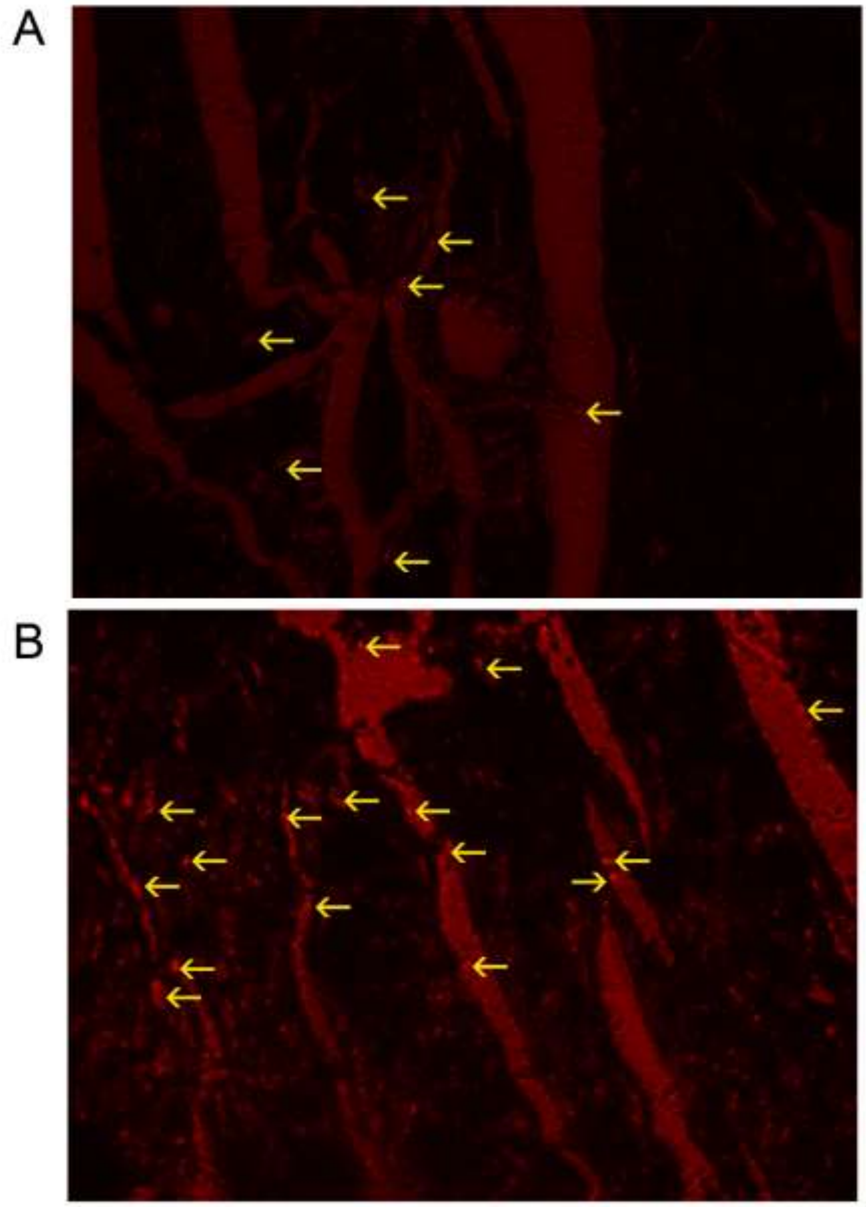

Figure 1. Supplementary, Fluorescence microscopy of MSCs (yellow arrows) labeled with immunostained with PKH26 fluorescent dye showing their survival in the myocardium one month after transplantation. 400×, A Diabetic rats treated with MSCs, B diabetic rats treated with MSCs + PIO.

Supplementary Figure 1

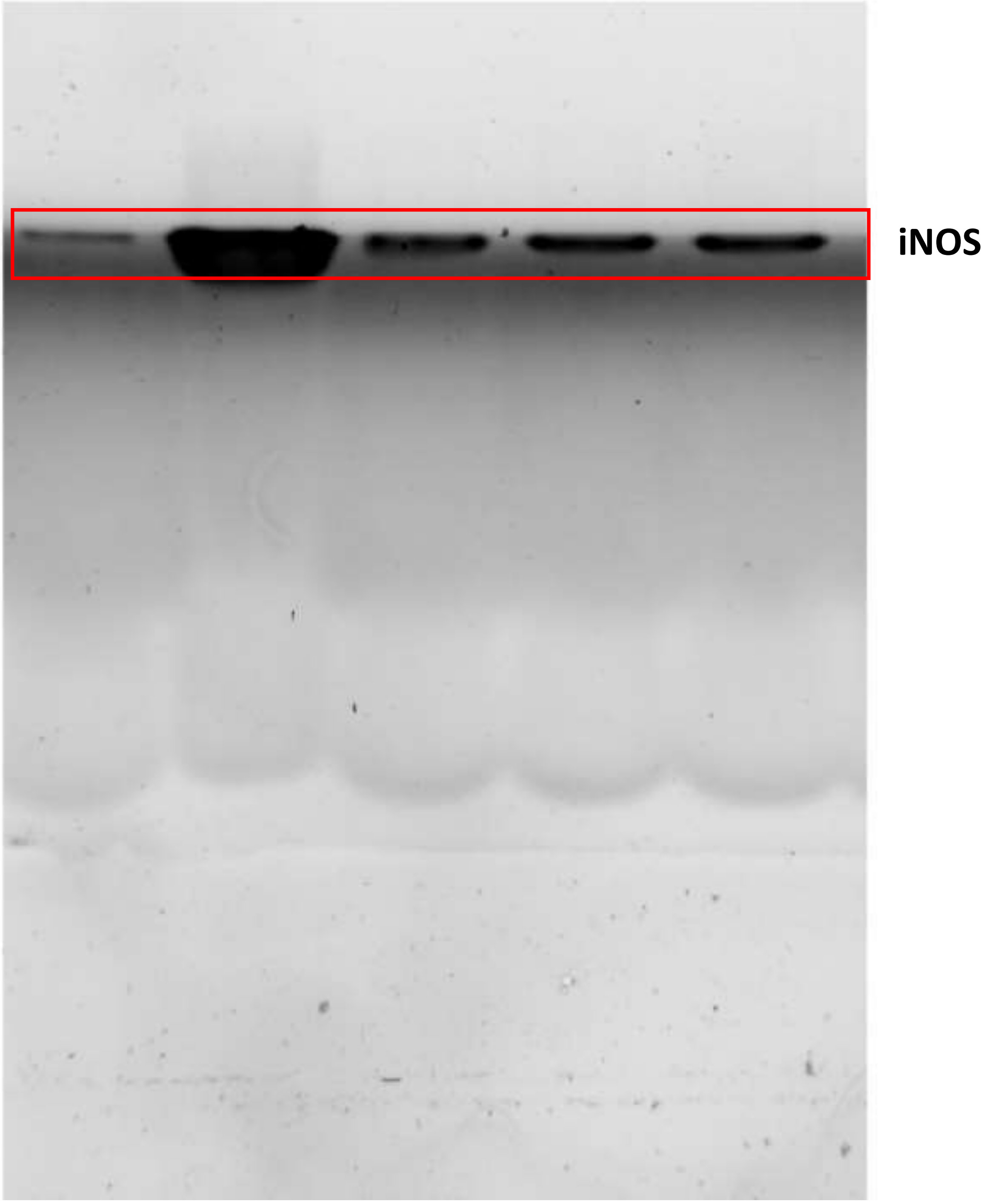
iNOS

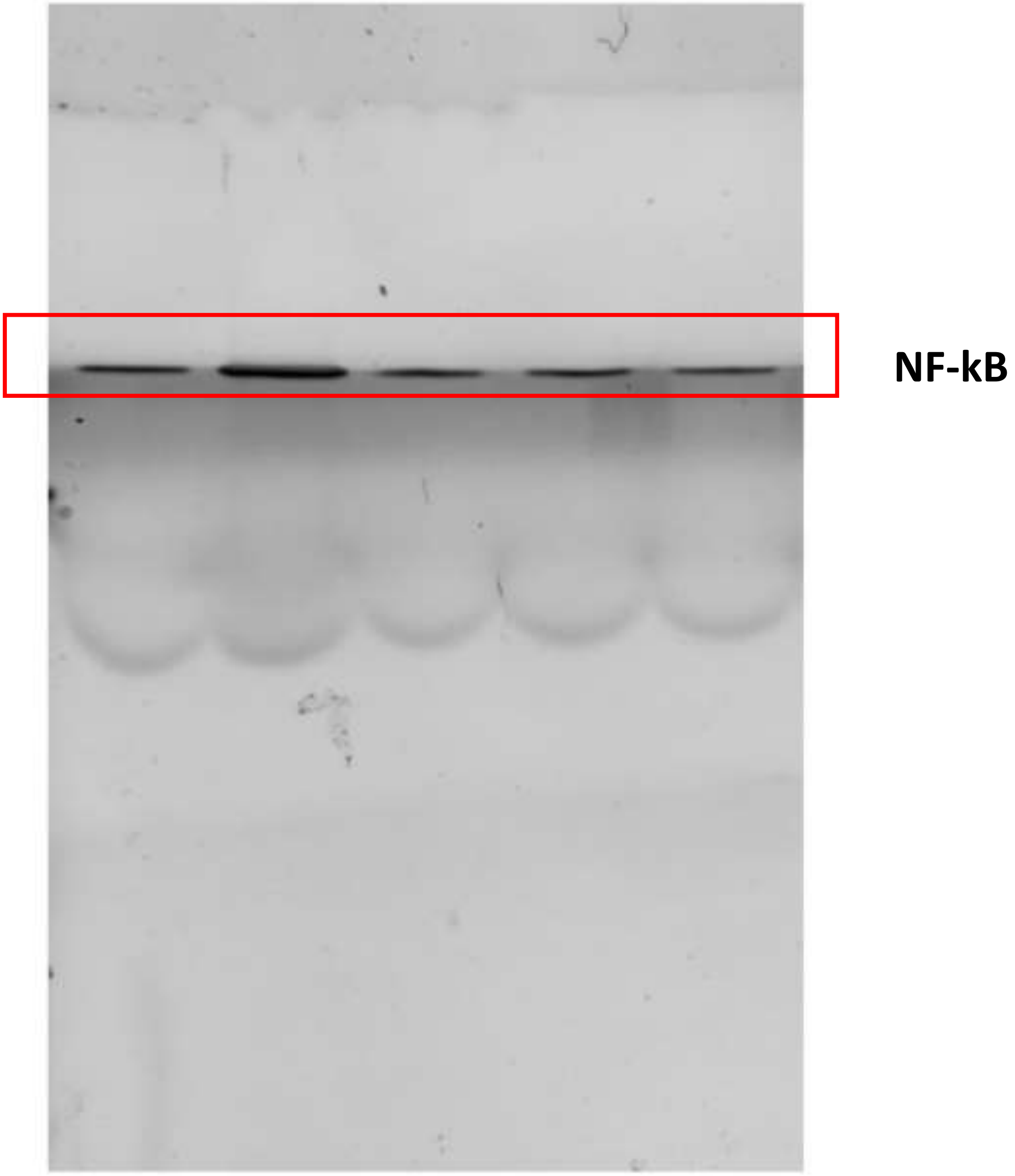

Supplementary Figure 2. The original blots of figure 5E.

Supplementary Figure 2

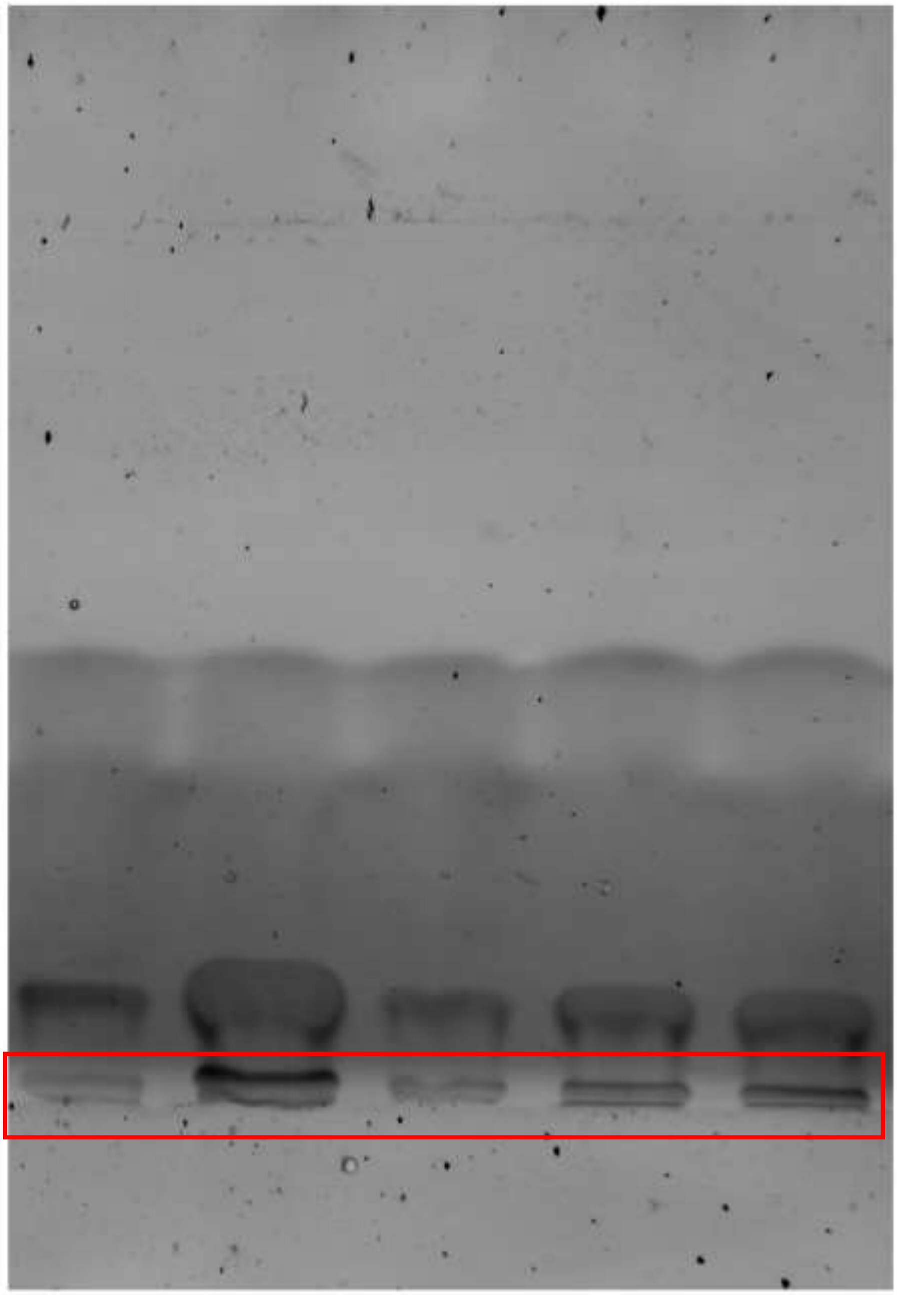
Cleaved Caspase

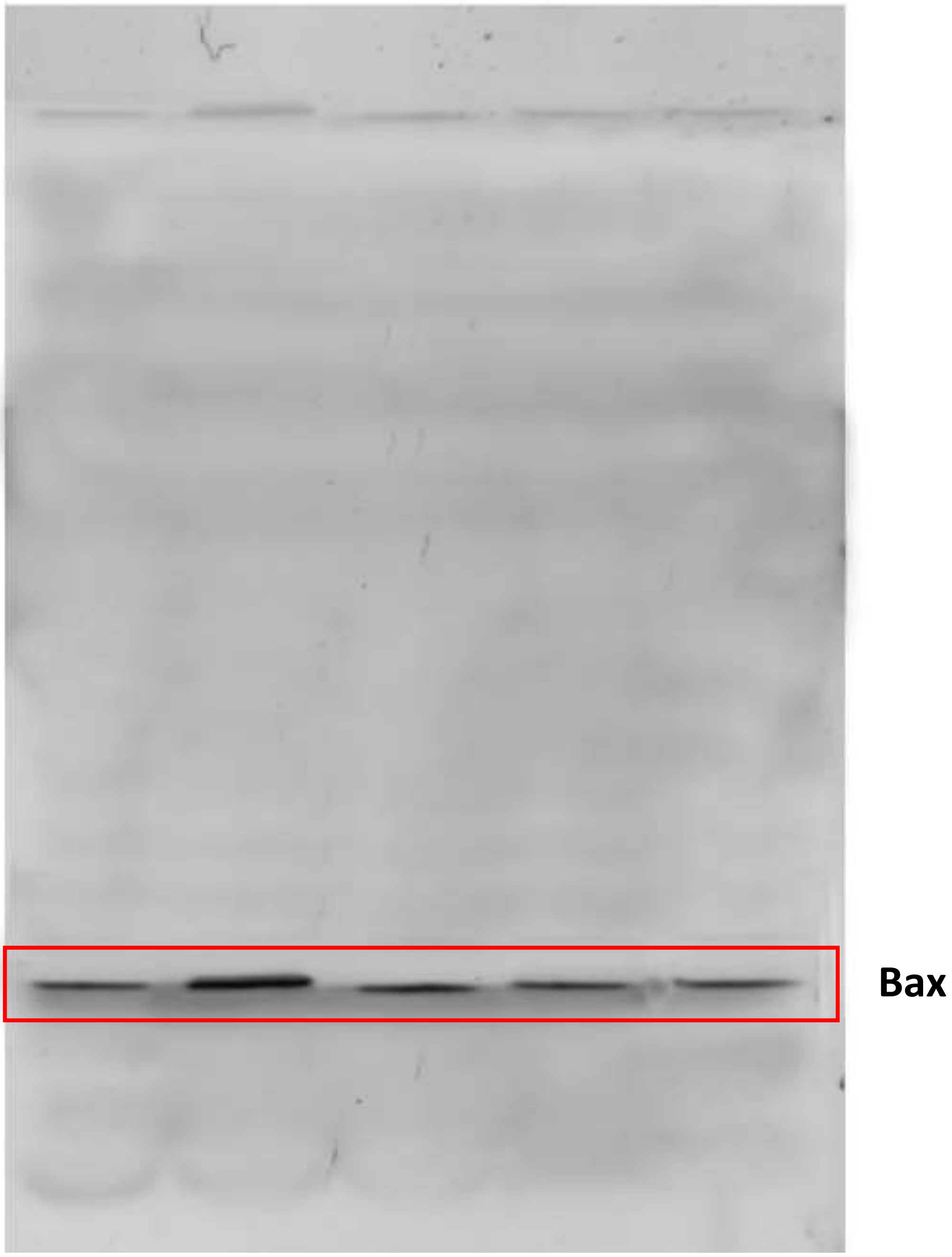
Bax

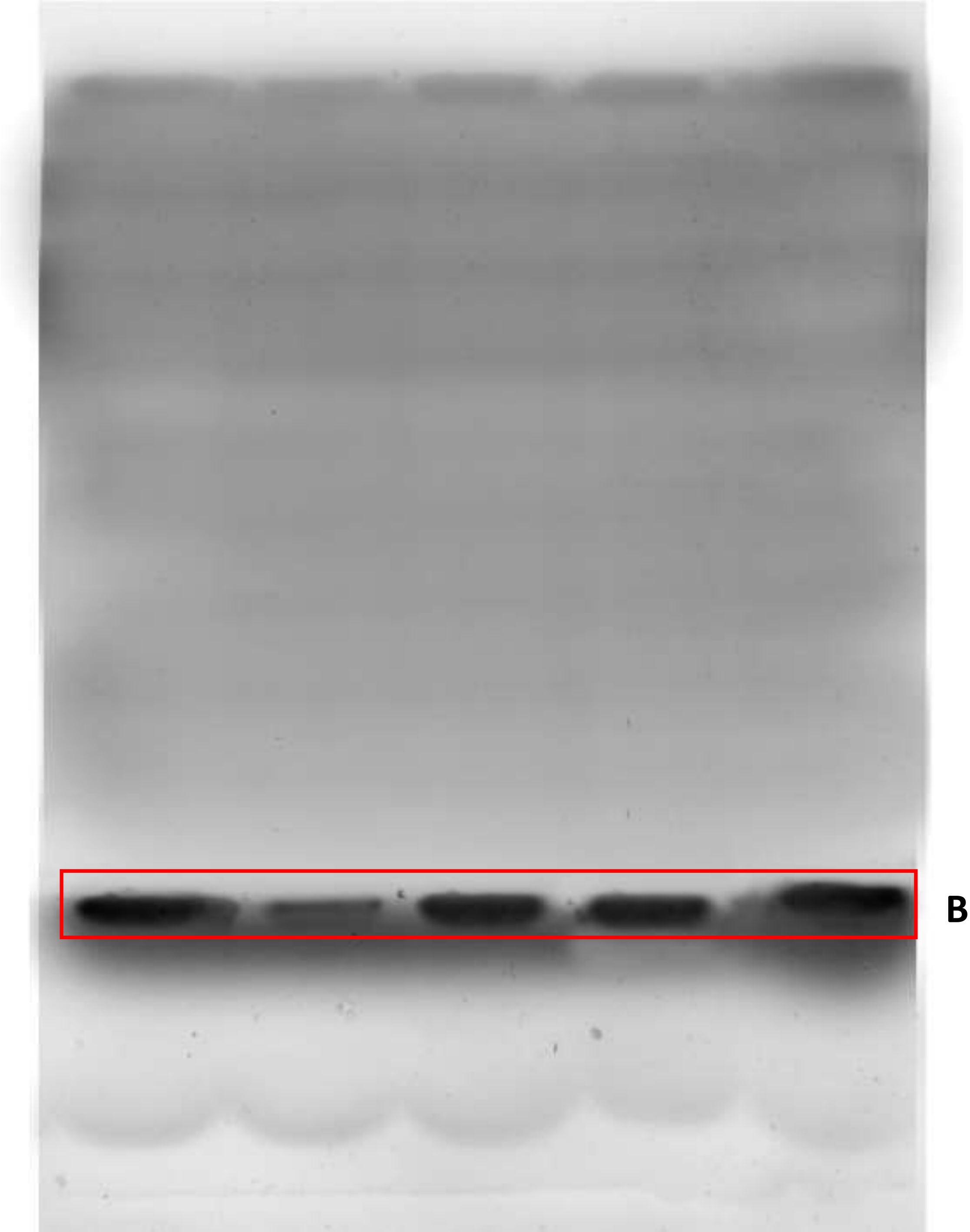

BCl-2

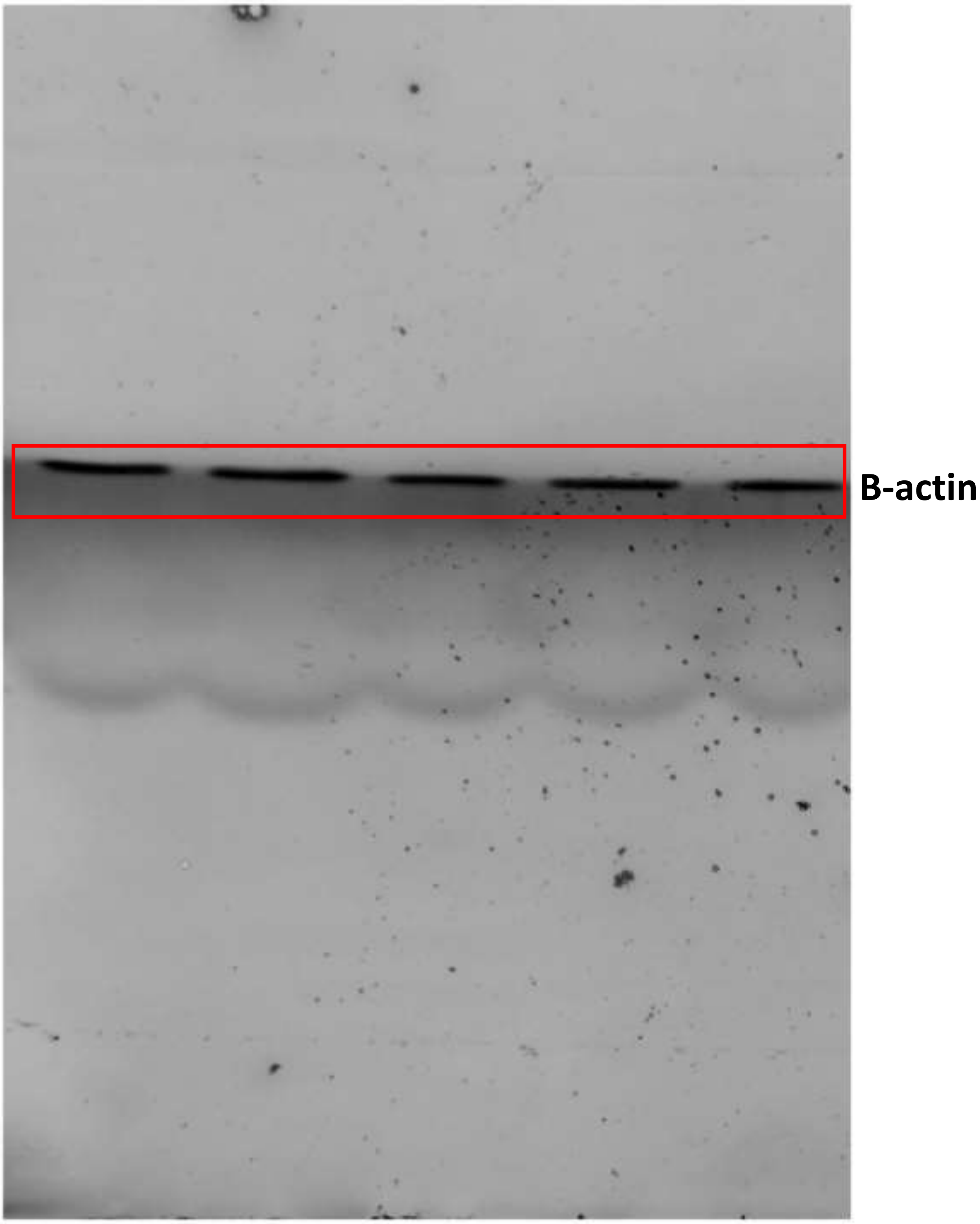

Supplementary Figure 3. The original blots of figure 6C.

Supplementary Figure 3
